# Population and landscape genomics provide insights into the adaptive genetic variation and future climate-induced vulnerability of the endangered tree species *Phoebe bournei*

**DOI:** 10.1093/hr/uhag165

**Published:** 2026-04-24

**Authors:** Junhong Zhang, Yan Liu, Xiange Hu, Li Wang, Ningning Fu, Xiaohua Ma, Wenjun Ma, Yuting Zhang, Xiao Han, Qi Yang, Zaikang Tong

**Affiliations:** State Key Laboratory for Development and Utilization of Forest Food Resources, Zhejiang A&F University, Hangzhou 311300, China; Zhejiang Key Laboratory of Forest Genetics and Breeding, Zhejiang A&F University, Hangzhou 311300, China; State Key Laboratory for Development and Utilization of Forest Food Resources, Zhejiang A&F University, Hangzhou 311300, China; Zhejiang Key Laboratory of Forest Genetics and Breeding, Zhejiang A&F University, Hangzhou 311300, China; State Key Laboratory for Development and Utilization of Forest Food Resources, Zhejiang A&F University, Hangzhou 311300, China; Zhejiang Key Laboratory of Forest Genetics and Breeding, Zhejiang A&F University, Hangzhou 311300, China; State Key Laboratory for Development and Utilization of Forest Food Resources, Zhejiang A&F University, Hangzhou 311300, China; Zhejiang Key Laboratory of Forest Genetics and Breeding, Zhejiang A&F University, Hangzhou 311300, China; State Key Laboratory for Development and Utilization of Forest Food Resources, Zhejiang A&F University, Hangzhou 311300, China; Zhejiang Key Laboratory of Forest Genetics and Breeding, Zhejiang A&F University, Hangzhou 311300, China; State Key Laboratory for Development and Utilization of Forest Food Resources, Zhejiang A&F University, Hangzhou 311300, China; State Key Laboratory of Tree Genetics and Breeding, Research Institute of Forestry, Chinese Academy of Forestry, Beijing 100000, China; State Key Laboratory for Development and Utilization of Forest Food Resources, Zhejiang A&F University, Hangzhou 311300, China; Zhejiang Key Laboratory of Forest Genetics and Breeding, Zhejiang A&F University, Hangzhou 311300, China; State Key Laboratory for Development and Utilization of Forest Food Resources, Zhejiang A&F University, Hangzhou 311300, China; Zhejiang Key Laboratory of Forest Genetics and Breeding, Zhejiang A&F University, Hangzhou 311300, China; State Key Laboratory for Development and Utilization of Forest Food Resources, Zhejiang A&F University, Hangzhou 311300, China; Zhejiang Key Laboratory of Forest Genetics and Breeding, Zhejiang A&F University, Hangzhou 311300, China; State Key Laboratory for Development and Utilization of Forest Food Resources, Zhejiang A&F University, Hangzhou 311300, China; Zhejiang Key Laboratory of Forest Genetics and Breeding, Zhejiang A&F University, Hangzhou 311300, China

## Abstract

Elucidating the genomic underpinnings of adaptive variation is highly important for the conservation, landscape application, and management of ornamental trees against the backdrop of global climate change. However, research on the genetic mechanisms underlying climate adaptation in *Phoebe bournei*—a near-threatened subtropical tree species endemic to China, which is endowed with exceptionally high ornamental and ecological value—remains scarce. Whole-genome resequencing was conducted on 362 individuals from 27 natural populations across the geographical range of the species. Genome-environment association analyses were employed to identify 1556 climate-associated variants and 167 candidate genes associated with temperature and precipitation variables. Through functional annotation and expression profiling, pivotal genes, including *TRX-M4* and *FBD1,* were identified as integral to drought and heat stress responses, with adaptive alleles displaying distinct geographic frequency distributions and significant phenotypic differentiation. Divergent evolutionary trajectories were deduced among populations, with southeastern populations distinguished by elevated genetic diversity and strong signatures of local adaptation. Nevertheless, projections derived from the Risk of Non-Adaptedness and gradient forest models suggest that these southeastern populations will face substantial genomic offset under future climate scenarios, signaling heightened vulnerability and the need for prioritized conservation and management. This study provides the first genome-wide perspective into the adaptive evolution of *P. bournei* and offers a robust foundation for its conservation and climate-resilient management.

## Introduction

Climate change, particularly alterations in temperature and precipitation regimes, is projected to emerge as a principal threat to global biodiversity. There is ample empirical evidence documenting climate-induced local extinctions among plant species [1, 2]. As sessile organisms, plants must adapt to changing environments through phenotypic plasticity or genetic variation, especially in trees with long growth cycles. Intraspecific variation in tolerance to climate change indicates that populations with high adaptive potential are expected to cope with changes in habitat suitability arising from climate change [3]. However, adaptive responses are not uniform across species [4], necessitating a deeper integration of genomic insights with population-level ecological responses to climate-driven environmental shifts.

Nanmu, particularly *Phoebe bournei*, holds profound ecological and cultural significance and has been historically revered as ‘imperial wood’ because of its exceptional durability and extensive use in royal architecture such as the Forbidden City [5–7]. However, despite this heritage, natural populations are rapidly declining because of anthropogenic disturbances and climate change, leading to its classification as ‘near threatened’ on the IUCN Red List. As a subtropical species, *P. bournei* is highly sensitive to drought and climatic fluctuations [8, 9]. Although recent propagation efforts have expanded its cultivation [10, 11], the reliance on limited genetic resources in plantations has undermined environmental adaptability and recovery potential [12, 13]. Given that its underlying genetic architecture remains poorly understood, integrating genomic insights with predictive modeling is urgently needed to inform adaptive conservation strategies.

However, accurate prediction of the suitable future distribution of tree species remains a significant challenge because of the inherent assumptions and uncertainties embedded in species distribution models [14–17]. Nevertheless, the use of statistical modeling approaches remain a scalable and effective strategy for assessing species’ climatic suitability. Genomic approaches such as assessment of the Risk of Non-Adaptedness (RONA) enable the quantification of population-level vulnerability by estimating the degree of mismatch between current allele frequencies and projected climatic conditions [18, 19]. Additionally, gradient forest models extend this framework by capturing nonlinear relationships between genetic variation and environmental gradients, thereby identifying key climatic drivers of local adaptation and predicting genomic vulnerability under future climate scenarios [4]. Together, these methods provide critical insights into the genomic basis of local adaptation and vulnerability, offering a powerful means to understand how climate change may reshape the geographic distribution of tree species and inform targeted conservation and management strategies [20].

In this study, we performed whole-genome re-sequencing of 362 individuals from 27 natural populations spanning the entire distribution range of *P. bournei* to characterize its genome-wide variation. Our specific objectives were to: (i) reconstruct the demographic history and population structure to understand the evolutionary forces shaping the current distribution of *P. bournei*; (ii) identify the genomic signatures of environmental associations and elucidate the key climatic drivers responsible for adaptive genetic variation; and (iii) predict genomic vulnerability under future climate scenarios to inform targeted conservation and management strategies for *P. bournei*.

## Results

### High-quality whole-genome resequencing reveals substantial genetic variation in *P. bournei*

The statistics of whole-genome resequencing are shown in Table S1. An average of 95.95% of the clean reads per sample were successfully mapped to the reference genome, with a mean sequencing depth of ~20× (Table S2). A total of 9.65 million single nucleotide polymorphisms (SNPs) were identified, of which 42.3%, 35.1%, 19.4%, and 3.1% were located in intergenic regions, introns, exons, and coding sequences (CDSs), respectively (Fig. S1). Additionally, 389 181 indels (≤50 bp) and 4288 structural variants (SVs) (>50 bp) were detected across the genome. A subset of 574 572 independent variants was subsequently generated by removing linkage disequilibrium (LD) for population structure inference.

### Low genetic differentiation across geographically distant populations

**Figure 1:**
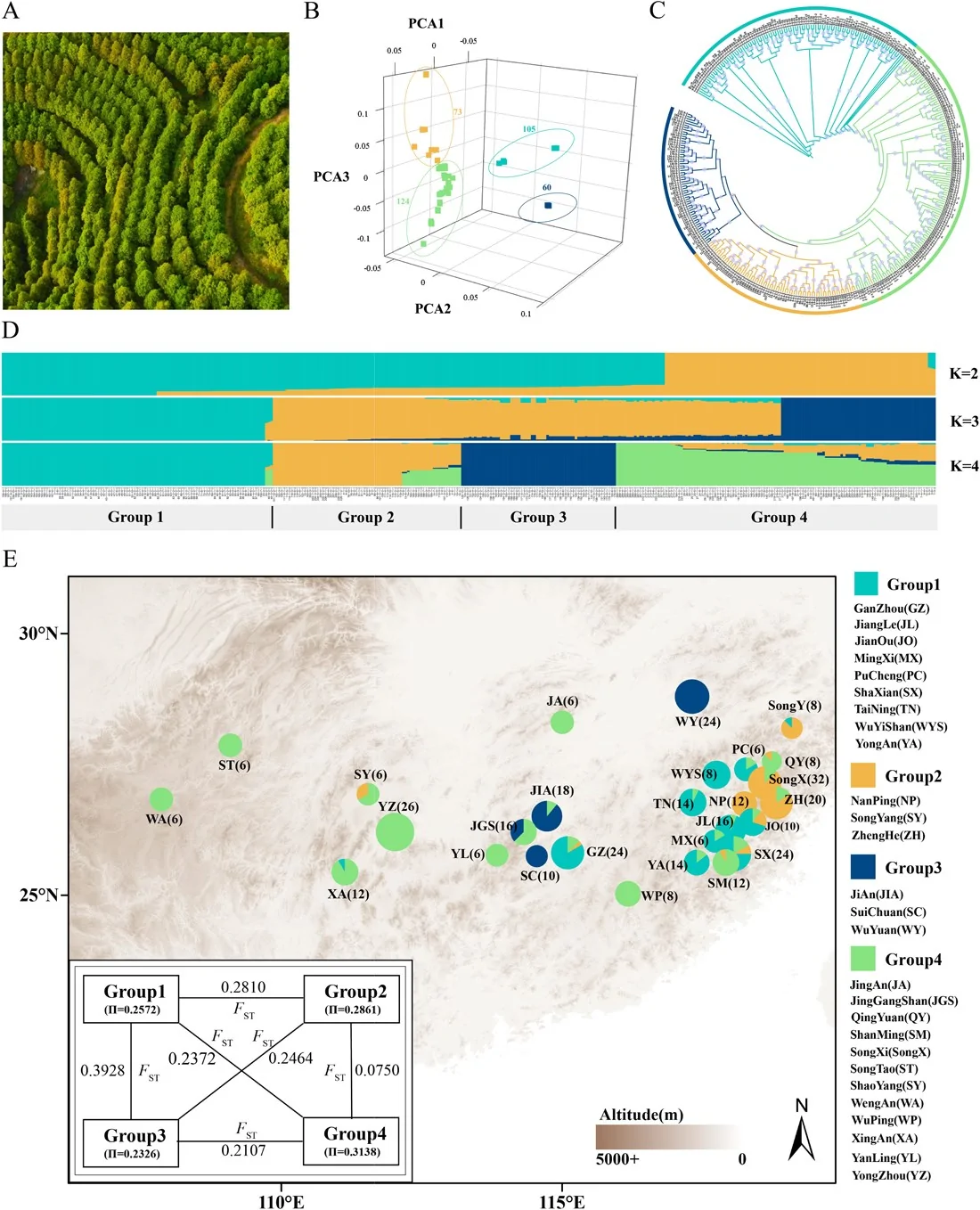
Population genomic variation and structure of *P. bournei*. (A) Photographs of the *P. bournei* germplasm bank. (B) 3D-PCA analysis of 362 individuals. (C) ML phylogenetic tree of 362 individuals. The bootstrap support values are indicated by transparent circles, with the circle size proportional to the support value. (D) Model-based population assignment using ADMIXTURE with *K* values ranging from 2 to 4. (E) Geographic distribution of 27 natural populations of *P. bournei*. The size of each pie chart is proportional to the population sample size (*N*) and the different segments indicate inferred ancestral components. The inset in the lower-left corner displays the nucleotide diversity (*π*) values for the four ancestral clusters and the pairwise *F*_ST_ values among the populations.

**Figure 2:**
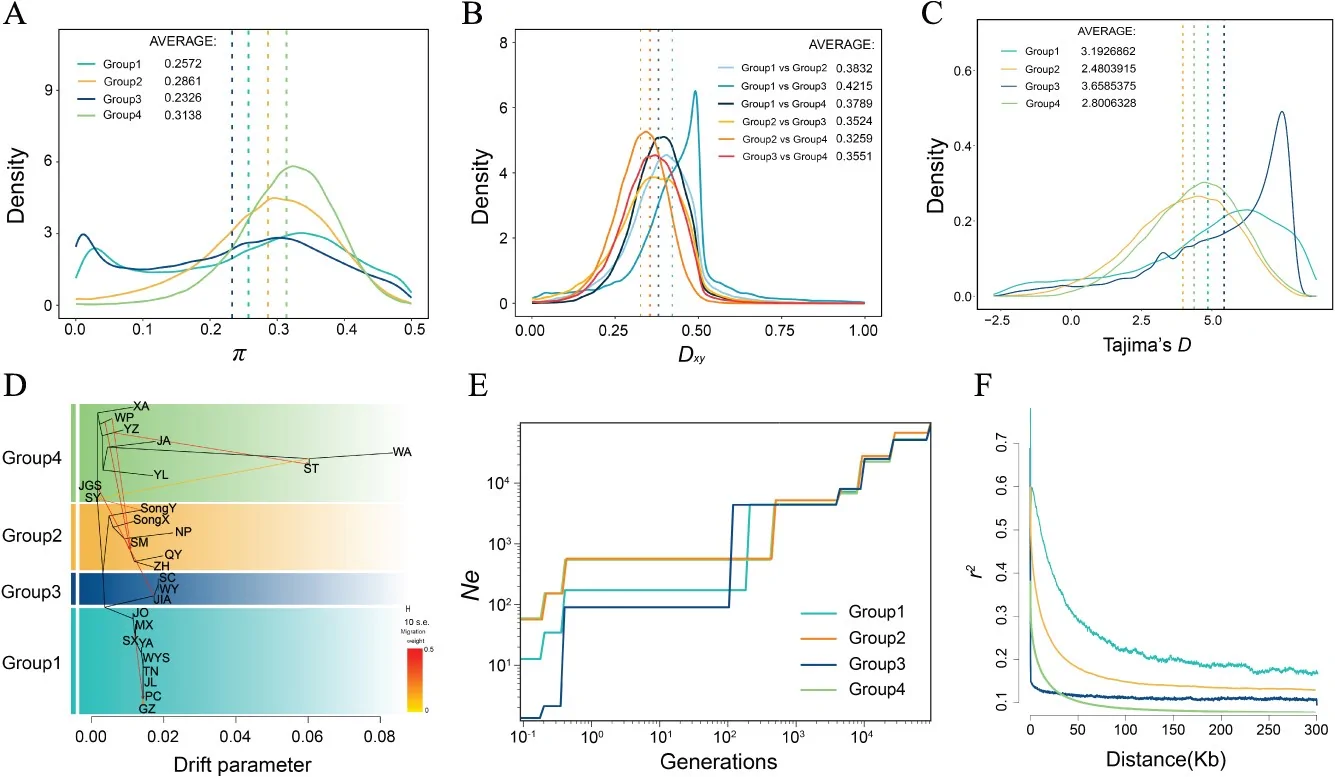
Genetic diversity, differentiation, and demographic history of *P. bournei* populations. (A) Genome-wide nucleotide diversity (*π*) calculated in 100-kb nonoverlapping windows for the four genetic groups. (B) Absolute nucleotide divergence (*D*_xy_) between group pairs, calculated in 100-kb nonoverlapping windows. Dashed lines indicate the genome-wide average values. (C) Distribution of Tajima’s *D* values for the four ancestral clusters. (D) Population relationships and gene flow among 27 populations inferred from genetic drift parameters. (E) Demographic history showing the temporal dynamics of the effective population size (*N_e_*) for the four clusters, inferred using SMC++. (F) LD decay curves for the four ancestral clusters.

Population structure analysis revealed four genetic clusters (*K* = 4). Three of these clusters were distributed primarily in the eastern region, whereas the range of Group 4 extended into the western part of the species distribution (Figs 1D and S2). Principal component analysis (PCA) and maximum likelihood (ML) analyses revealed similar results (Fig. 1B and C). Group 4 exhibited significant isolation-by-distance (IBD) signals, whereas Groups 1–3 showed weak IBD patterns (Fig. S3A-D). Notably, despite their substantial geographic separation, Group 2 and Group 4 displayed the lowest pairwise differentiation, with an average *F*_ST_ of 0.0750 (Fig. 1E) and the lowest absolute divergence (*D*_xy_) among all pairs (Fig. 2B). In contrast, the greatest differentiation was observed between Group 1 and Group 3 (*F*_ST_ = 0.3928). In terms of genetic diversity, Group 4 presented the highest nucleotide diversity (*π* = 0.3138), whereas Group 3 presented the lowest (*π* = 0.2326) (Figs 2A and S4A). Furthermore, TreeMix analysis revealed historical migration events connecting the lineages, providing explicit evidence of gene flow between Group 2 and Group 4 (Fig. 2D).

### Demographic history and LD

The results of the historical population dynamics of *P. bournei* indicated that all four genetic groups experienced two distinct periods of population contraction (Fig. 2E). Following these events, the population sizes generally stabilized. Groups 2 and 4 exhibited highly similar demographic trajectories, whereas Groups 1 and 3 displayed divergent patterns. Group 3 experienced a severe bottleneck during the recent contraction, with its Ne plummeting from ~100 individuals to near zero, indicating a potential local extinction event. These demographic inferences were corroborated by positive Tajima’s *D* values across all the groups, reflecting historical population contractions, with Group 3 showing the highest *D* value, which is consistent with an elevated proportion of intermediate-frequency alleles (Fig. 2C). LD analysis revealed that the *r*^2^ decreased below 0.2 at ~20 kb across all groups. Group 3 exhibited the fastest LD decay rate, indicating extensive linkage breakdown, likely resulting from past demographic perturbations (Figs 2F and S4B).

### Adaptive variants in *P. bournei* are strongly shaped by environmental factors

To elucidate the role of environmental factors in shaping adaptive genetic variation in *P. bournei*, we employed two genome-environment association (GEA) approaches—latent factor mixed model (LFMM) and redundancy analysis (RDA)—to analyze associations between genotypes and 19 bioclimatic variables (Bio1–Bio11: temperature-related; Bio12–Bio19: precipitation-related). Combined LFMM and RDA analyses revealed 1556 core variant sites strongly associated with those variables, comprising 1091 SNPs, 463 indels, and two SVs linked to 167 candidate genes. Functional annotation revealed that five SNPs were located within coding exons, 54 variants (41 SNPs and 13 indels) occurred in the 2 kb upstream regulatory regions, 36 variants (22 SNPs and 14 indels) occurred in 2 kb downstream regions, and 125 variants (81 SNPs and 44 indels) were located within intronic regions (Fig. S5).

The adaptive variants exhibited significant IBD and isolation-by-environment (IBE) patterns, particularly in Group 4, whereas the neutral variants showed no significant patterns of IBD or IBE (Fig. S6A, B). Group 2 displayed a notable IBE pattern (Figs S3 and S7A-D). Partial RDA revealed that climate, geography, and population structure collectively explained 75% of the genetic variation in adaptive variants, with climate alone accounting for 16% (vs 12% for neutral variants) when controlling for geography and population structure. Population structure explained 17% of the adaptive variation when controlling for climate and geography (Fig. S8A, B).

### Association of promoter variation with climatic adaptation in *P. bournei*

**Figure 3:**
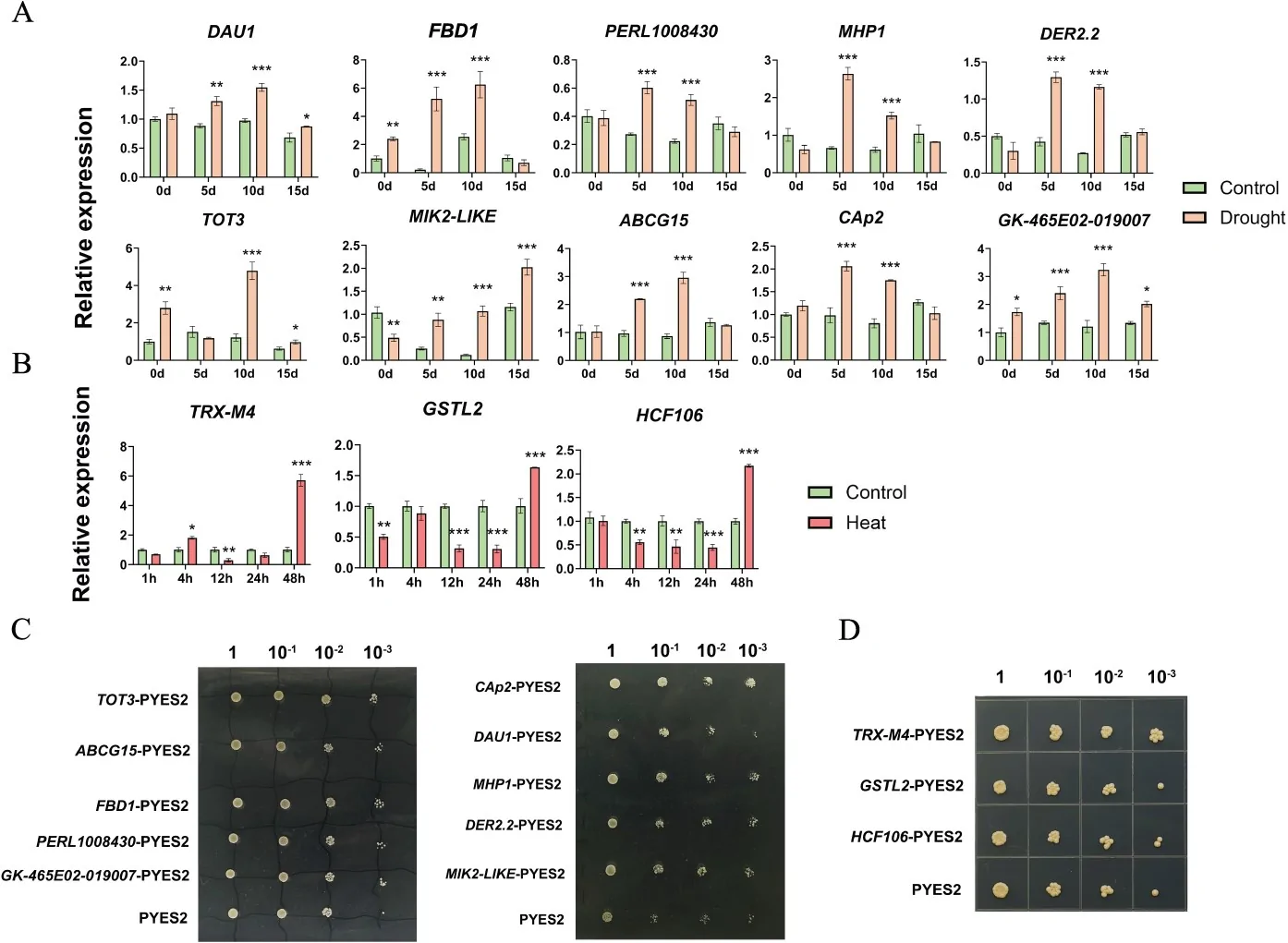
Functional validation of candidate genes under drought and heat stress. (A) qRT-PCR analysis of the 10-candidate drought-tolerance genes in *P. bournei* leaves subjected to natural drought treatment at 0, 5, 10, and 15 days. (B) qRT-PCR analysis of three heat tolerance candidate genes in *P. bournei* leaves after high-temperature treatment (40°C) for 1, 4, 12, 24, and 48 hours. (C, D) Growth assays of *S. cerevisiae* INVSc1 strains overexpressing candidate genes under PEG-induced drought stress (C) and elevated-temperature stress (D).

Among the 167 candidate genes identified for climatic adaptation in *P. bournei*, 13 genes with promoter region variants were selected for detailed investigation of their potential roles in drought and heat stress responses (Table S3). Under natural drought conditions, ten precipitation-associated genes were significantly upregulated between days 5 and 10; *FBD1* (an *F-box* family gene) showed a 6-fold increase in expression, *TOT3* and four other genes increased by 3–4-fold, and the remaining genes increased by 2–3-fold (Fig. 3A). After 48 hours of heat stress, the expression of three temperature-associated genes increased, with the expression of *TRX-M4* (a chloroplast M-type thioredoxin homolog) increasing 5.7-fold (Fig. 3B). Functional association analyses revealed a significant correlation between these gene variants and stress responses. For example, under simulated drought conditions, most variant genotypes exhibited enhanced environmental adaptation potential, and the variation in *TRX-M4* was also associated with improved heat tolerance (Fig. 3C and D).

**Figure 4:**
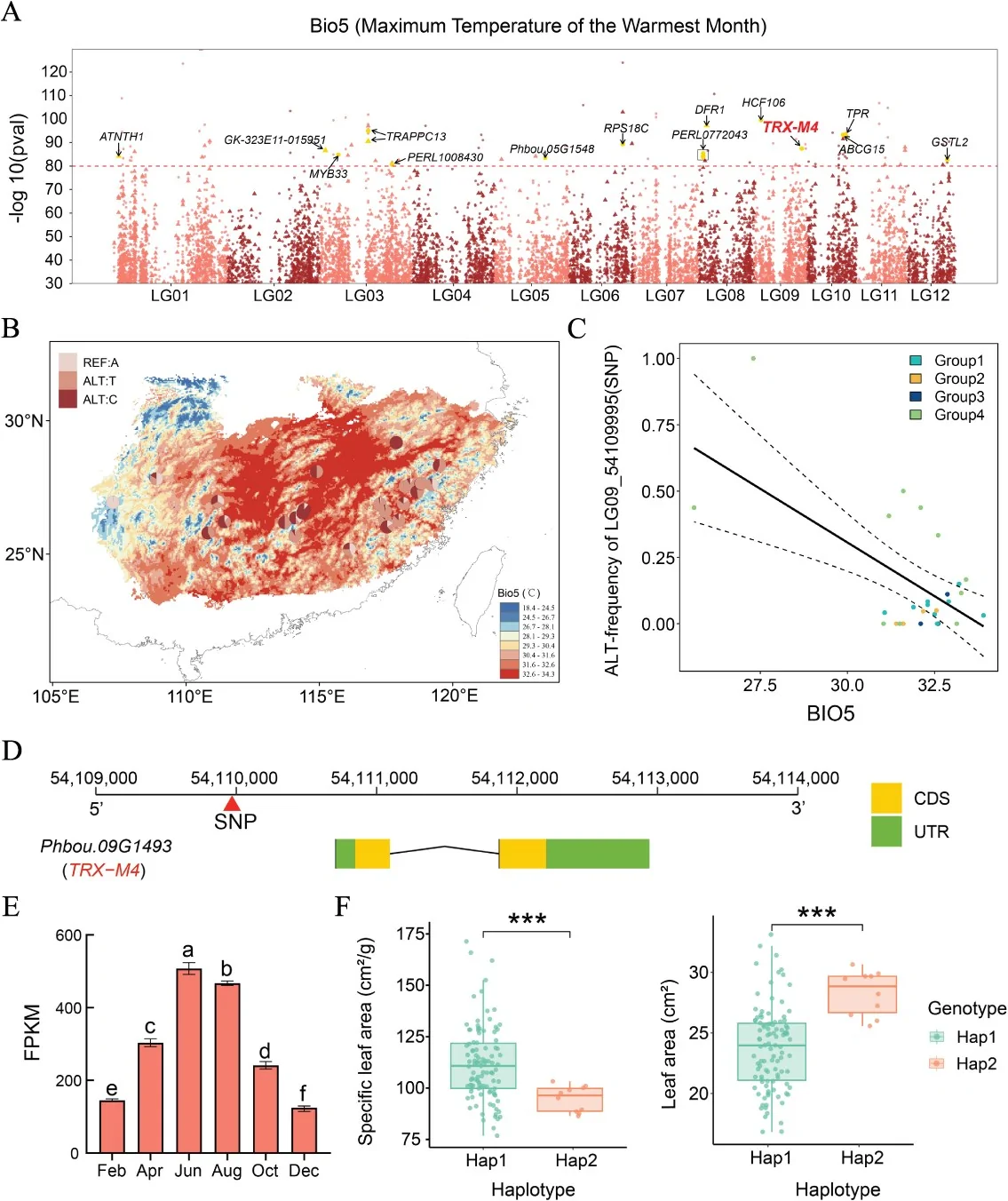
Genome-wide screening for loci linked to local environmental adaptation. (A) Manhattan plot of the maximum temperature of the warmest month (Bio5), with the candidate genes highlighted. (B) Geographic allele-frequency map of the Bio5-associated adaptive SNP at LG09:54109995 across 27 populations; the map shading reflects spatial variation in the climatic variable. (C) Linear regression of allele frequency against Bio5 values across the 27 populations, dashed lines indicate the 95% confidence intervals. (D) Gene model of the *P. bournei TRX-M4* homolog, with triangles indicating the position of the adaptive site. (E) Bar chart of transcript levels (FPKM) for the *TRX-M4* homolog in leaves collected throughout the year. Different letters above the bars denote statistically significant differences. (F) Association analysis of the two homozygous haplotypes with leaf area and SLA. The error bars represent the SD. Asterisks (***) indicate statistically significant differences at *P* < .001 (Student’s *t*-test).

A combined analysis of temperature and moisture sensor (TMS) monitoring and meteorological data confirmed that the study site experienced severe soil water deficit (volumetric water content: 8%–10.5%) and heat stress during the summer of 2022 (Fig. S9A, B). To investigate the ecological consequences of genotypic variation under these conditions, we assessed critical tolerance traits—including leaf area, specific leaf area (SLA), and the summer-to-spring shoot ratio—across the entire resequenced population (Table S4). An allele frequency analysis of *TRX-M4* (associated with Bio5: maximum temperature of the hottest month) revealed that allele A was predominant in cooler regions, whereas alleles *T* and *C* were predominant in warmer regions (T mostly in Groups 1 and 2; C mostly in Groups 3 and 4; Fig. 4A–C). *TRX-M4* (with the variable locus LG09:54109995) is a homolog of *AT3G15360* and belongs to the chloroplast M-type thioredoxin family, which plays a vital role in the redox processes of leaf photosynthesis metabolism [21]. This variant, which is located in the promoter region of *TRX-M4* (Fig. 4D), coincided with peak summer expression (Fig. 4E) and showed no evidence of positive selection (|iHS| = 0.21) (Fig. S10A). Haplotype analysis revealed that Hap2 (harboring the A allele; frequency = 0.112) harbors an ‘AAAG’ motif associated with chlorophyll biosynthesis and the heat stress response [22]. In contrast, this motif is absent in the T-allele haplotype Hap1 (frequency = 0.387; Fig. S11A). Phenotypically, compared to individuals that were homozygous for Hap2, individuals that were homozygous for Hap1 had significantly smaller leaf areas but significantly greater SLAs (Fig. 4F).

**Figure 5:**
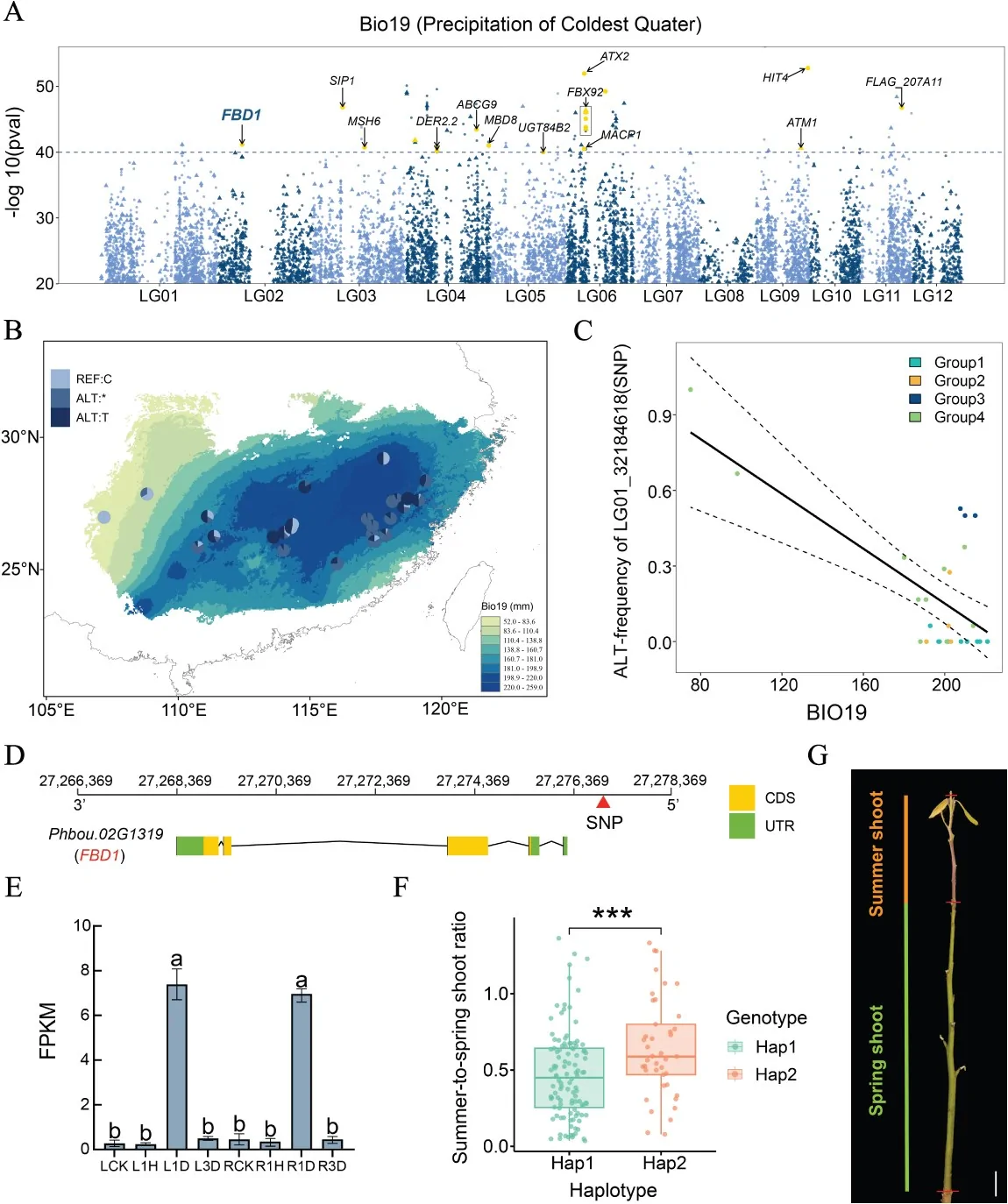
Genome-wide screening of the loci associated with local environmental adaptation. (A) Manhattan plot of the precipitation in the coldest month (Bio19), with the candidate genes highlighted . (B) Geographic allele-frequency map of the Bio19-associated adaptive SNP at LG02:27276701 across 27 populations; the map shading represents spatial variation in the climatic factor. (C) Linear regression of allele frequency against Bio19 precipitation values across the 27 populations, with dashed lines indicating 95% confidence intervals. (D) Gene structure of the *P. bournei FBD1* homolog, with triangles indicating the adaptive site. (E) Bar chart of the transcript abundance (FPKM) of the *FBD1* homologs in leaf and root tissues under PEG-induced drought stress. Different letters denote statistically significant differences. (F) Association analysis of the two homozygous haplotypes with the summer-to-spring shoot ratio. The error bars represent the SD. Asterisks (***) indicate statistically significant differences at *P* < .001 (Student’s *t*-test). (G) Morphology of *P. bournei* shoots showing the lengths of summer and spring shoots. Scale bar = 2 cm.

An allele frequency analysis of *Phbou.02G1319* (associated with Bio19: precipitation of the coldest quarter) revealed that populations in regions with higher precipitation during the coldest quarter (winter) tended to have more T or missing alleles (*), with T predominantly fixed in Groups 2 and 4, while missing (*) alleles were more prevalent in Group 1. Conversely, regions with lower precipitation showed a higher frequency of allele C (Fig. 5A–C). *Phbou.02G1319* (with the variable locus LG02:27276701) is a homolog of *Arabidopsis FBD1* (*AT1G13570*), which belongs to the F-box family and plays a role in plant stress tolerance processes such as drought [23], heat stress [24], and cold stress [25]. This variant is located in the promoter region of *Phbou.02G1319* (Fig. 5D), and it coincided with significant expression in leaves and roots after 1 day of simulated drought treatment (Fig. 5E), whereas extended haplotype homozygosity (EHH) analysis indicated no evidence of positive selection at this locus (|iHS| value = 0.43) (Fig. S10B). Haplotype analysis revealed that Hap1 (which carries the deletion allele; freq. = 0.315) features the *cis*-regulatory motif ‘TTGAC’—a canonical binding site for WRKY transcription factors involved in plant stress responses [26]. In contrast, this motif is absent in Hap2 (which carries the T allele; freq. = 0.113) (Fig. S11B). Phenotypically, Hap1 individuals presented a significantly lower summer-to-spring shoot ratio compared to Hap2 individuals (Fig. 5F and G).

### Climate-driven habitat loss and adaptive vulnerability in *P. bournei* populations

**Figure 6:**
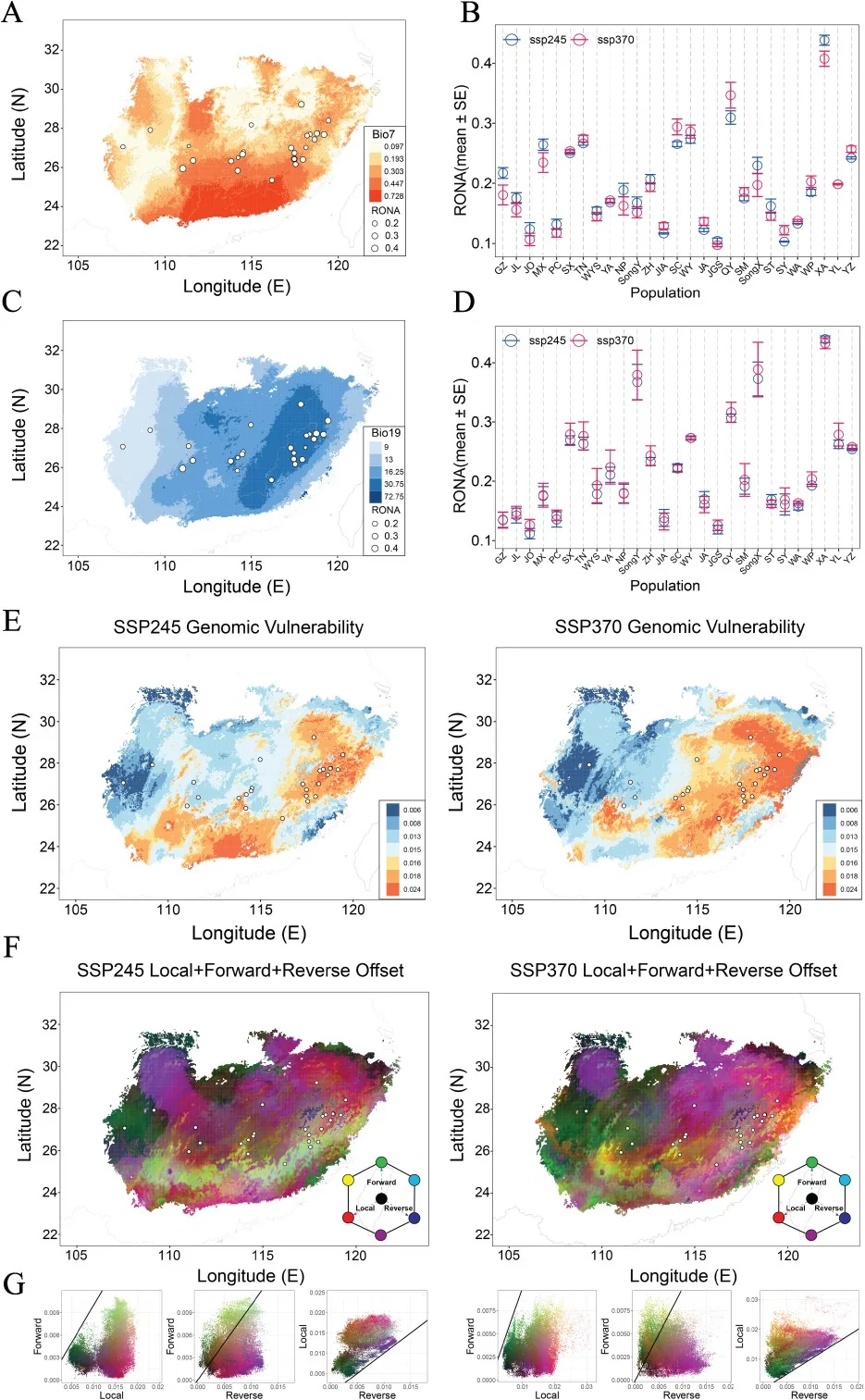
RONA and predicted genetic offsets of *P. bournei* populations to future climatic conditions under SSP126 and SSP370 for 2061–2080. (A and C) Average RONA values of the four climate models for the 27 populations under the SSP245 climate scenario for 2041–2060 (A: Bio7; C: Bio19). The raster shading on the map indicate the trends of projected future climate change. Regions with darker shading are predicted to experience more changes in the respective climate variables. The size of the circles on the map indicates the average RONA values for 27 natural populations. (B and D) Comparison of the mean RONA values under two different climate scenarios (SSP245 and SSP370) for 2041–2060 for the 27 populations for Bio7 (B) and Bio19 (D). The error bars represent the SEs of the average RONA values calculated from the four different climate models for Bio7 and Bio19. (E) Genetic offset of the 27 sampled populations, with a gradient scale indicating increasing offset values. (F) RGB composite maps of local, forward, and reverse genetic offsets within the suitable habitat of *P. bournei* under SSP245 and SSP370. Brighter cells (closer to white) along each axis denote relatively higher offset values, whereas darker cells (closer to black) denote relatively lower values. (G) Bivariate scatter plots comparing genetic offset values between the SSP245 and SSP370 scenarios, with the 1:1 reference line indicated.

Ecological niche modeling (ENM) revealed that *P. bournei* underwent progressive southward contraction from the northern regions of the Yangtze River, resulting in significant loss over high-suitability areas (Fig. S12A–D). To evaluate future climate-induced vulnerability, we integrated adaptive genetic variation with climate projections using RONA and Gradient Forest (GF) analyses. Six bioclimatic variables with low multicollinearity (|*r*| < 0.6)—temperature-related (Bio3: isothermality, Bio7: annual temperature range, and Bio8: mean temperature of wettest quarter) and precipitation-related (Bio12: annual precipitation, Bio17: precipitation of the driest quarter, and Bio19: precipitation of the coldest quarter)—were selected for RDA (Figs S13 and S14). RONA exhibited high correlations across 27 populations, with minimal variation between the SSP245 and SSP370 scenarios (Figs S15 and S16A, B). Southeastern populations displayed elevated RONA values under SSP245 for 2041–2060, indicating a reduced adaptive capacity to future temperature and precipitation extremes (Fig. 6A–D). The GF analysis, which incorporated core climate variables, also revealed greater genetic divergence in southeastern populations, suggesting increased extinction risk (Figs 6E and S17). By incorporating migration dynamics, we calculated the local, forward, and reverse genetic offsets; southeastern populations consistently showing higher values across migration distances (100, 250, 500, and 1000 km and unrestricted) and pollution models, particularly for forward offset (Fig. 6F and G).

## Discussion

In this study, we conducted a systematic genome-wide analysis to elucidate the genetic differentiation, adaptive variation, and future climate responses of *P. bournei*. Our discussion focuses on three core insights: (i) historical demography: the counterintuitive genetic affinity between geographically distant populations (Group 2 and Group 4) was driven by postglacial range expansion rather than recent long-distance dispersal; (ii) adaptive mechanism: adaptive genetic variation along climatic gradients is characterized predominantly by polygenic regulatory variations involving key genes such as *TRX-M4* and *FBD1*; and (iii) future risk: southeastern populations, despite their high genetic diversity, face the highest risk of maladaptation under future warming scenarios, necessitating genomics-informed conservation strategies.

### Demographic history and postglacial expansion shaped the genetic landscape of *P. bournei*

Although we delineated four genetic clusters across the full distribution range of *P. bournei* using a combination of three independent methods (Fig. 1), these groups exhibited a pronounced spatial pattern. Strong genetic differentiation was observed in most pairwise comparisons among the four groups, with the notable exception of Group 2 and Group 4, which displayed high genetic similarity (Fig. 2). Notably, despite their substantial geographic separation, with Group 2 situated along the southeastern coast and Group 4 in the northwestern inland, these two groups showed high genetic similarity. This ‘geographically distant but genetically similar’ pattern implies a recent divergence or extensive historical gene flow between them (Fig. 2D) [27]. Considering the evolutionary history of the species, we hypothesize that Group 2 may have originated from Group 4 via a recent range expansion that was likely associated with postglacial climatic amelioration [28]. This scenario is consistent with postglacial expansion patterns reported in other subtropical evergreen tree species in East Asia [29, 30]. Furthermore, given the historical status of *P. bournei* as ‘imperial wood’, anthropogenic dispersal cannot be entirely ruled out as a contributing factor [5–7] . Human-mediated germplasm transfer for cultivation could have accelerated gene flow between these distant regions, reinforcing the observed genetic affinity [31, 32]. Thus, we conclude that postglacial expansion, not recent gene flow, is the key driver of the observed ‘distant yet similar’ genetic pattern. *P. bournei* is likely structured into three primary ancestral lineages. Group 1 and Group 3 exhibit relatively independent genetic structures, whereas Group 2 likely represents a secondary lineage derived from Group 4 that migrated eastward. With increasing environmental heterogeneity, Group 2 appears to be evolving into a geographically distinct subpopulation with potential local adaptations (Figs 1E and 2) [33, 34].

### Climatic gradients shape the landscape of putative adaptive genetic variants in *P. bournei*

Identifying the genetic basis of how organisms respond to environmental heterogeneity is crucial for understanding their evolutionary potential [34, 35]. In this study, 1556 core adaptive loci responsive to 19 climatic variables were identified in *P. bournei*. Notably, in Group 4, adaptive loci displayed pronounced patterns of IBE (Figs S3 and S7), suggesting that environmental gradients, alongside geographic barriers, contribute to the spatial structuring of genetic variation. These patterns align with theoretical expectations that heterogeneous habitats may promote the maintenance of spatially varying genotypes [36]. Furthermore, the spatial distribution of allele frequencies in specific candidate genes provided insights into how *P. bournei* potentially responds to climatic factors [37, 38]. We observed significant correlations between the allele frequencies of functional genes and key climatic variables (Figs 4 and 5). For instance, the redox-related gene *TRX-M4* strongly correlated with temperature variables (Bio5 and Bio10). The A allele is predominant in cooler regions, whereas the *T* and *C* alleles were more frequent in warmer regions (Fig. 4B). Similarly, *FBD1* was significantly associated with precipitation (Bio19), with distinct allele frequency shifts observed between wet and dry habitats (Fig. 5B). These findings indicate that under environmental stress, adaptive alleles may undergo divergent selection, leading to their accumulation in different populations, which is consistent with the model of local adaptive differentiation driven by climatic gradient selection [33, 34, 39]. Common garden surveys during the 2022 drought verified that phenotypic differentiation in terms of leaf morphology and shoot ratios represents constitutive genetic adaptation (Figs 4F and 5F). We propose a dual regulatory strategy driven by promoter variants: leaf morphology provides a static structural defense, whereas shoot ratios enable dynamic drought avoidance via phenological plasticity. Thus, natural selection targets specific regulatory elements to synchronize a plant’s static defenses with its dynamic response capabilities [40].

Although strong positive selection signals were not detected at most adaptive loci (Fig. S10A and B), the presence of natural selection is not negated. These variations may be governed by polygenic adaptation [41, 42]. In fact, long-lived species such as forest trees, with their extended generation intervals and slow reproductive cycles, may adapt to environmental changes through gradual shifts in the allele frequencies of multiple moderate-effect variants [43]. The abundant functional variations identified in promoter or regulatory regions (Table S3) further corroborate this hypothesis. These regulatory variations modulate gene expression intensity, tissue specificity, or stress responses without directly altering protein structure, representing a robust molecular strategy for plant environmental adaptation [44, 45]. Collectively, these findings underscore the pivotal role of *cis*-regulatory evolution in shaping the adaptive landscape of *P. bournei*, enabling a sophisticated integration of structural robustness and physiological plasticity to navigate climatic heterogeneity.

### Climatic history and genomic offset highlight adaptive risk in southeastern *P. bournei* populations

In a review of quaternary climate evolution, both last interglacial (LIG) and last glacial maximum (LGM) profoundly influenced the historical distribution and genetic structure of plant populations [46]. ENM suggested that *P. bournei* experienced a significantly broader suitable habitat during the warm and humid LIG, with its northern distribution boundary shifting considerably and suitable habitats widely extending across the Huang-Huai Plain southward and the hilly regions north of the Yangtze River (Fig. S12A). In contrast, during the LGM, dramatic climatic changes led to dramatic range contraction [47, 48], with high-suitability areas confined to the southeastern hilly zone between the Nanling Mountains and the Wuling-Dabie-Yellow mountain systems (Fig. S12B). The region’s complex terrain and diverse microenvironments likely served as climatic refugia [28, 49], buffering environmental extremes and preserving critical genetic diversity and adaptive potential for *P. bournei*. These simulated historical distribution patterns are consistent with archaeological findings and historical records of Nanmu coffin usage [50]. At present, the Earth is in the interglacial period (the Holocene, beginning 11 ka BP) following the last glaciation; however, anthropogenic global warming is occurring at a pace far exceeding historical natural rhythms, posing unprecedented challenges to plant distributions and adaptive patterns [51].

Therefore, in-depth, genome-wide variation analysis serves as a critical foundation for developing predictive models of species responses to future climate change, thereby providing essential support for the formulation of scientifically informed conservation and adaptation strategies [37]. Our prediction results indicate that during the period from 2041 to 2060, southeastern regions of the species distribution, particularly Zhejiang and Fujian, will experience substantial climate-driven adaptive pressures (Fig. 6). The current adaptive genotypes in these populations may be insufficient to cope with rapidly changing climatic conditions [38, 52]. Multidimensional based assessments on forward, reverse, and local genomic offsets further indicate that even with moderate natural migration, these populations are unlikely to locate fully suitable climatic habitats (Fig. 6F), thereby increasing the long-term risk of persistence [53]. Given the relatively high level of genetic diversity in this southeastern region (Figs 1E and 2), conservation strategies should integrate both forward and reverse offset metrics to guide targeted actions based on population-specific adaptive capacity and habitat characteristics. Measures such as assisted migration to climatically stable western regions and the introduction of favorable genotypes from western populations—adapted to future warmer and drier conditions—may enhance climate resilience in the southeast [54]. Furthermore, the establishment of a germplasm repository covering the entire geographic range of *P. bournei* will enable the application of breeding-based approaches to improve vulnerable populations genetically, thereby enhancing resilience to future climate change while safeguarding existing genetic diversity. In summary, the integration of genomic offset modeling highlights the critical vulnerability of southeastern populations, underscoring the urgent need for assisted migration and germplasm preservation.

### Limitations and future perspectives

While this study systematically assessed the adaptive evolutionary patterns of *P. bournei* at a large scale, several limitations warrant consideration. First, the practical utility of genomic offset models for natural populations remains uncertain [16]. Notably, phenotypic plasticity was not explicitly considered in adaptive capacity assessments, potentially leading to an underestimation of resilience [54]. Second, the genotype–phenotype associations were primarily validated through heterologous expression in yeast. Further validation through haplotype-phenotype association analyses and transgenic functional assays is required to confirm these causal relationships. Third, adaptive analyses focus exclusively on climatic variables and overlook the potential contributions of edaphic factors and soil microbial communities to local adaptation [55, 56]. Consequently, interpretations of adaptive patterns should be cautious. Future research should integrate multiomics datasets, incorporate cross-environmental factors (e.g. climate–plant–microbiome interactions), and establish a species-specific functional genomics pipeline to elucidate adaptation mechanisms comprehensively and inform conservation strategies for endangered subtropical tree species under climate change.

## Conclusion

This study integrated population genomics with ecological niche modeling to elucidate the complex interplay of demographic history and adaptive genetic variation in shaping the genetic landscape of *P. bournei*. Our findings reveal that the current population structure of this species—particularly the high genetic affinity between geographically distant southeastern and northwestern lineages—was likely driven by postglacial range expansion and historical gene flow. We further elucidated the pivotal role of *cis*-regulatory evolution in shaping the adaptive landscape, specifically by coordinating structural robustness with physiological plasticity to navigate heterogeneous climatic environments. In this context, key candidate genes such as *TRX-M4* and *FBD1* were identified as central mediators of responses to temperature and precipitation gradients. Crucially, genomic offset assessments indicate that southeastern populations face a heightened risk of maladaptation under future climate warming scenarios. These insights underscore the urgency of implementing genomics-informed conservation strategies—including the establishment of comprehensive germplasm repositories and assisted migration programs—to increase the long-term resilience of this valuable subtropical tree species against rapid environmental change.

## Materials and methods

### Selection of resequencing samples

The re-sequenced materials were uniformly cultivated at the national germplasm resource bank of *P. bournei*, which is located in Qingyuan County, Lishui city, Zhejiang Province (119.096°E, 27.635°N), an area with a subtropical monsoon climate. This region has an annual precipitation of 2010 mm and the mean annual temperature is 17.4°C (https://www.tianqi24.com/lishui/history2022.html). Afforestation of the germplasm resource bank was conducted in early 2014, covering an area of six hectares. Two-year-old seedlings were planted at a spacing of 3 ×3 m. The experimental seedlings originated from 181 healthy seed samples collected across 27 counties in six provinces of China (Table S4, Fig. 1A). For resequencing, we selected two individuals that displayed the greatest divergence from each family. Notably, seeds were harvested from superior mother trees with a minimum spatial distance of 100 m to ensure genetic independence. All 10-year-old *P. bournei* individuals currently maintained in the plantation were included as sampling targets for whole-genome resequencing in this study. Healthy, fully expanded mature leaves were collected from the middle canopy layer. Owing to the limited population size, all accessible individuals were incorporated into the analysis, encompassing 181 families with two individuals sampled from each family, resulting in a total of 362 individuals.

### Genomic DNA extraction and resequencing

High-quality genomic DNA was isolated from *P. bournei* leaf tissue using a modified CTAB protocol [57]. Libraries meeting the quality criteria were sequenced on an Illumina NOVAseq 6000 platform using 150 bp paired-end reads to a depth of ~20× (~20 Gb per sample). Raw image data were converted to sequence reads by base calling, and adapter sequences, low-quality bases, and reads containing undetermined nucleotides were removed to produce high-quality clean reads. All library preparation and sequencing procedures were performed by Novogene Bioinformatics Technology Co., Ltd. (Beijing, China).

### SNP calling and repeat masking

Clean reads were aligned to the *P. bournei* reference genome [6] using BWA-MEM (http://bio-bwa.sourceforge.net/). Picard (https://broadinstitute.github.io/picard/) was then employed to mark duplicates, sort reads, and construct BAM indices. Alignment statistics for each sample were obtained with samtools flagstat. Variant calling was performed with GATK (https://software.broadinstitute.org/gatk/). Per-sample BAMs were processed through HaplotypeCaller in GVCF mode to generate individual GVCF files, which were then combined via GenotypeGVCFs into a multisample VCF. SNPs were extracted and hard-filtered using the following criteria: QD < 2.0, MQ < 40.0, FS > 60.0, SOR > 3.0, MQRankSum < −12.5, and ReadPosRankSum < −8.0. The functional annotation of all the filtered SNPs was performed using ANNOVAR [58] and SnpEff (https://pcingola.github.io/SnpEff/download/) together with the *P. bournei* genome annotation. We constructed two variant datasets using PLINK (https://www.cog-genomics.org/plink2/) to accommodate specific analytical needs. First, applying basic filters (−geno 0.2, −maf 0.05, −biallelic-only) yielded a ‘High-quality variant dataset’ (10 015 612 variants) for environmental association and nucleotide diversity analyses. Second, pruning this set for LD (−indep-pairwise 50 10 0.2) produced an ‘LD-pruned dataset’ (574 572 variants) specifically for population structure inference.

### Population structure analysis

PLINK (v1.90b6.21) [59] was used with the parameters ‘indep-pairwise 50 10 0.2’ to extract a set of LD-pruned SNPs with a minor allele frequency (MAF) > 10%. ADMIXTURE (v1.3.0) [60] was applied with default parameters to explore the population genetic structure, with the number of clusters (*K*) set from 1 to 10. PCA was conducted using PLINK (v1.90b6.21) on the pruned SNPs, and a 3D PCA plot was generated using the R package *plot3D* to visualize the results [61]. Additionally, an ML phylogenetic tree was constructed using IQ-TREE 2 [62], with the best-fit substitution model selected by ModelFinder and branch support assessed via 1000 ultrafast bootstrap replicates, and the tree was visualized using iTOL (https://itol.embl.de/).

### Genetic diversity, LD, and demographic history analysis

The sequence diversity (*π*) and *D*_xy_ nucleotide diversity were calculated using the pixy program (v1.2.7.beta1) [63]. Nonoverlapping windows of 25, 10, 1, and 2 kb were used for the four ancestral groups. Additionally, Tajima’s *D* statistics were calculated using VCFtools (v0.1.16) [64] within the same nonoverlapping windows for each group based on the complete SNP dataset. To estimate and compare LD patterns among groups, the squared correlation coefficient (*r*^2^) between pairwise SNPs with an MAF > 0.1 was calculated using PopLDdecay [65] and averaged across the genome. Gene flow events among populations were inferred using the Python package *Treemix* [66]. Residual fitting was performed to identify the model with the minimum residual value, which provided an estimate of the most likely number of gene flow events. Historical changes in the effective population size (*Ne*) of *P. bournei* were inferred using SMC++ (v1.15.2) [67]. A per-generation mutation rate of 3.7×10^−8^ was applied, and the inferred *Ne* trajectories over a time span of 10^2^ to 10^5^ generations were visualized using the smc++ plot function to illustrate temporal fluctuations in population size.

### Identification of environment-associated genetic variants

Two different approaches were employed to identify environment-associated variants (SNPs, indels, and SVs) across the whole genome. Only common variants with an MAF > 0.1 were retained. First, a univariate LFMM [68] was applied using the R package *LEA* v3.3.294 [69] to search for associations between allele frequencies and the 19 BioCLIM environmental variables. For each environmental variable, five independent Markov chain Monte Carlo (MCMC) runs were conducted, with 5000 iterations used as burn-in followed by 10 000 iterations. The *P*-values from all five runs were then averaged for each variant and adjusted for multiple tests using a false discovery rate (FDR) correction of 5% as the significance cutoff. Second, RDA was performed using the R package *vegan* [70], in which multivariate climate variables served as the explanatory variables and the allele frequencies of genetic variants were treated as the response variables to identify loci strongly associated with environmental gradients [38, 71].

Mantel and partial Mantel tests were separately used to test for associations between *F*_ST_ (*F*_ST_/1-*F*_ST_) and IBD and IBE distance (after geographic distance was accounting for), with significance being determined using 9999 permutations in the R package *vegan*, where environmental distance was represented by the Euclidean distance of all scaled environmental variables [72]. In addition, a partial RDA was used to quantify the relative contributions of geography, population structure, and the environment to the proportion of adaptive and neutral genetic variation. For the two RDA models, the population allele frequencies of adaptive and neutral variants were used as the response variables. The significance of the explanatory variables was assessed using 9999 permutations with the function anova.cca of the *vegan* R package.

### Screening of candidate genes associated with temperature and moisture conditions

The environment-associated variants jointly identified by LFMM and RDA were intersected, and their classification and positional metadata were curated and annotated using the R packages *tidyr* and *dplyr* [73]. Manhattan plots depicting variant-environment associations were generated using *ggplot2* [74]. The allele frequencies of each variant across climatic gradients were calculated and visualized as pie charts at their corresponding geographic coordinates using *ggplot2*, *scatterpie*, and *stringr*. Gene structure models with annotated CDSs, untranslated regions (UTRs), and variant loci were constructed using *ggh4x*, *ggbio*, *GenomicRanges*, and *ggplot2*. Finally, functional inference was performed by integrating transcriptomic data from polyethylene glycol (PEG)-induced drought-treated leaf and root tissues [75] and monthly leaf fragments per kilobase of transcript per million mapped reads (FPKM) expression profiles. The EHH pattern was assessed for a selected set of strongly associated variants, and the standardized iHS across the genome for common variants was calculated using the R package *rehh* [76].

### Haplotype analysis and *cis*-regulatory element analysis

Haplotype phasing and LD block visualization across the target genomic region were performed using LDBlockShow v1.40 [77]. The analysis employed the BlockType haplotype to output haplotype structures, LDMethod *r*^2^ to compute pairwise SNP *r*^2^ values, MAF 0.05 to filter out low-frequency alleles, and Region chrX:start-end to define the exact coordinates. Haplotype-specific sequence fragments were then extracted from the reference genome and submitted to PlantCARE (https://bioinformatics.psb.ugent.be/webtools/plantcare/html/) for *cis*-regulatory element prediction.

### Cloning and sequencing alignment of the promoter fragments of candidate genes

For each homozygous haplotype, three individuals from distinct families were selected. DNA fragments encompassing 500 bp upstream and downstream of the target variant site were cloned and sequenced to validate the accuracy of the identified variants. All sequencing procedures were performed by Hangzhou Youkang Biotechnology Co., Ltd. (Hangzhou, China).

### Stress treatment and expression analysis by RT-qPCR

The 1.5-year-old *P. bournei* ‘Wuyuan No. 8’ seedlings were used for the drought and heat stress treatments. For the drought treatment, irrigation was withheld for 15 days, with a well-watered group maintained as the control. Leaf tissues were sampled every 5 days. In the heat treatment, the seedlings were transferred to 40°C, while a parallel set remained at 24°C as the control, and the leaves were harvested at 1, 4, 12, 24, and 48 h after-exposure. Each treatment consisted of five biological replicates, and at each time point, 3–5 fully expanded leaves from the shoot apex of each seedling were collected for subsequent analyses.

Total ribonucleic acid (RNA) was extracted using the M5 Plant RNeasy Complex Mini Kit (Mei5 Biotechnology, Beijing). *PbEF1α* was used as the internal reference gene [75], and the quantitative primers for the candidate genes highly associated with adaptive loci are listed in Table S5. RT-qPCR was performed using ChamQ SYBR Color qPCR Master Mix (Vazyme, Nanjing) on a real-time PCR system, and relative expression levels were calculated via the 2^–ΔΔCt^ method [78]. Statistical analyses were conducted using SPSS v26.0. One-way analysis of variance (ANOVA) was performed to assess significance at the 95% confidence level (*P* < .05). For comparisons between two independent groups, two-tailed Student’s *t* tests were applied, with the assumption that the data satisfied normality (Shapiro–Wilk test) and homogeneity of variance (Levene’s test). Graphs of the results were generated using GraphPad Prism 9.

### Yeast ectopic expression analysis and stress tolerance assays

Yeast ectopic expression analysis and stress treatments were performed according to Fu *et al.* [79], with minor modifications. The gene-specific amplification primers used are listed in Table S6. Yeast INVSc1 cells containing recombinant plasmids and empty vector plasmids were subjected to 30% PEG 6000-simulated drought and 35°C heat stress (with 30°C as the control) for 36 hours to verify the tolerance of heterologously transformed yeast to drought and heat stress.

### Measurement of traits related to extreme heat and drought stress

To evaluate the impact of extreme heatwave and drought events during the summer of 2022, soil moisture conditions and key stress-adaptive traits were assessed in late September. Data on the monthly mean maximum temperature and monthly total precipitation in Lishui city for 2022 were obtained from the Environmental Meteorology Data Platform (http://eia-data.com/). Soil moisture status was characterized using a hybrid approach combining continuous TMS in-situ monitoring (recording at 7 cm depth every 15 min) [80]. Concurrently, key phenotypic traits— including leaf morphology (leaf area and SLA) and shoot growth patterns (lengths of summer and spring shoots) —were investigated.

### Ecological niche modeling

ENM was performed using Maxent (v3.4.4) and 19 current bioclimatic variables. Climate data were obtained from WorldClim (https://worldclim.org/data/worldclim21.html) with a spatial resolution of 30 seconds, covering LIG (129–116 ka BP), LGM (26.5–19 ka BP), and current (1970–2000) periods. In addition to the 362 individuals sampled from 27 natural populations in this study, 28 additional spatially independent occurrence records were selected from the Chinese Virtual Herbarium (https://www.cvh.ac.cn/) and the Global Biodiversity Information Facility (https://www.gbif.org/zh/) for Maxent analysis. The default parameters were used in Maxent, and environmental variables with a Spearman correlation coefficient > 0.6 were excluded to avoid multicollinearity. On the basis of the habitat suitability outputs from the Maxent model, the ASCII data were imported into ArcGIS for spatial visualization. The base map (Gs(2019)1651) was obtained from the Ministry of Natural Resources of China (http://bzdt.ch.mnr.gov.cn/).

### Genomic offset assessment

For each sampling site, environmental data for 19 bioclimatic variables (BioCLIM) were obtained from the WorldClim CMIP6 dataset [81] for two future periods (2021–2040 and 2041–2060), encompassing four global climate models: BCC-CSM2-MR, ACCESS-CM2, CMCC-ESM2, and GISS-E2–1-G, with a spatial resolution of 2.5 arc-minutes. Each dataset included projections under two shared socioeconomic pathways (SSP245 and SSP370). The relative importance of all 19 environmental variables and their pairwise correlations were comprehensively assessed, and key variables with pairwise correlation coefficients |*r*| < 0.6 were ultimately selected for subsequent analyses. The RONA method [19] was used to quantify the theoretical mean allele frequency shifts required for populations to adapt to future climate scenarios. The consistency of the RONA estimates across the four climate models was assessed using two-tailed Spearman correlation tests with 9999 permutations; the correlation coefficients and significance levels were reported in a results matrix. The RONA values were then calculated across all loci, populations, and climate variables for each climate model and time frame.

To complement the RONA approach, a nonparametric machine learning method, GF [4], was employed to model the genome-wide offset across the distribution range of *P. bournei*. GF models were constructed for each climate model using six selected noncollinear climatic predictors. Genomic offset was defined as the Euclidean distance between the current and projected genomic compositions and was visualized in ArcGIS 10.8. Additionally, to account for dispersal limitations, three types of genomic offset were calculated: local offset, forward offset, and reverse offset. Sensitivity analyses were performed for forward offset under varying maximum dispersal distances (100, 250, 500, 1000 km, and unlimited), providing insights into the influence of migration constraints on the adaptive potential of populations [38].

## Supplementary Material

Web_Material_uhag165

## Data Availability

All data sets are present in the paper and/or the Supplementary Materials. All resequencing data for 362 individuals in the present study have been deposited in the China National GeneBank Sequence Archive (CNSA) with accession number CNP0007867. Source data are provided with this paper. All scripts used in this study will be available at https://github.com/liuy12345671-commits/liu_repository/tree/main/Evolutionary-genomics-of-Phoebe-bournei upon publication. The base map of China (Gs(2019)1651) was obtained from the Ministry of Natural Resources of China (http://bzdt.ch.mnr.gov.cn/).
