# Supplementary material for: Population and landscape genomics provide insights into the adaptive genetic variation and future climate-induced vulnerability of the endangered tree species Phoebe bournei
Source: Hortic Res. 2026 Apr 24;13(9):uhag165. doi: 10.1093/hr/uhag165 (PMC13532409; doi:10.1093/hr/uhag165)
Supplement: Supplementary file 1 [file web_material_uhag165.zip › Fig. S1.pdf]

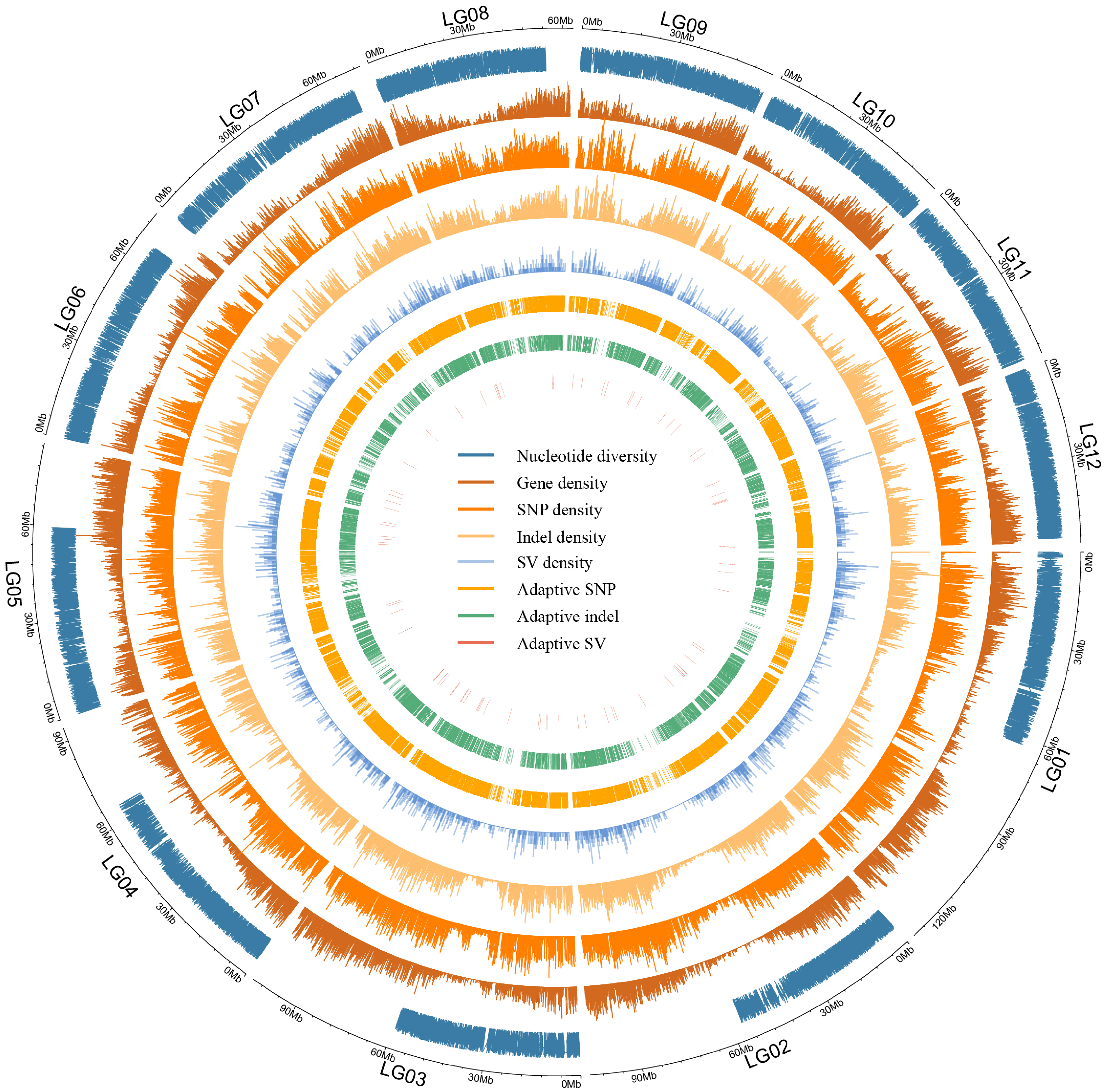

Fig. S1. Circos plot of genome-wide variation and adaptive loci distribution in *P. bournei*. The outermost ring shows linkage groups LG01–LG12 with physical coordinates (Mb). From outer to inner, the rings represent: nucleotide diversity (π), gene density, SNP density, InDel density, and SV density. The three inner rings display identified adaptive SNPs, adaptive InDels, and adaptive SVs. The height or length of each bar reflects the local abundance or distribution of the corresponding feature.
