# Supplementary material for: Population and landscape genomics provide insights into the adaptive genetic variation and future climate-induced vulnerability of the endangered tree species Phoebe bournei
Source: Hortic Res. 2026 Apr 24;13(9):uhag165. doi: 10.1093/hr/uhag165 (PMC13532409; doi:10.1093/hr/uhag165)
Supplement: Supplementary file 1 [file web_material_uhag165.zip › Fig. S11.pdf]

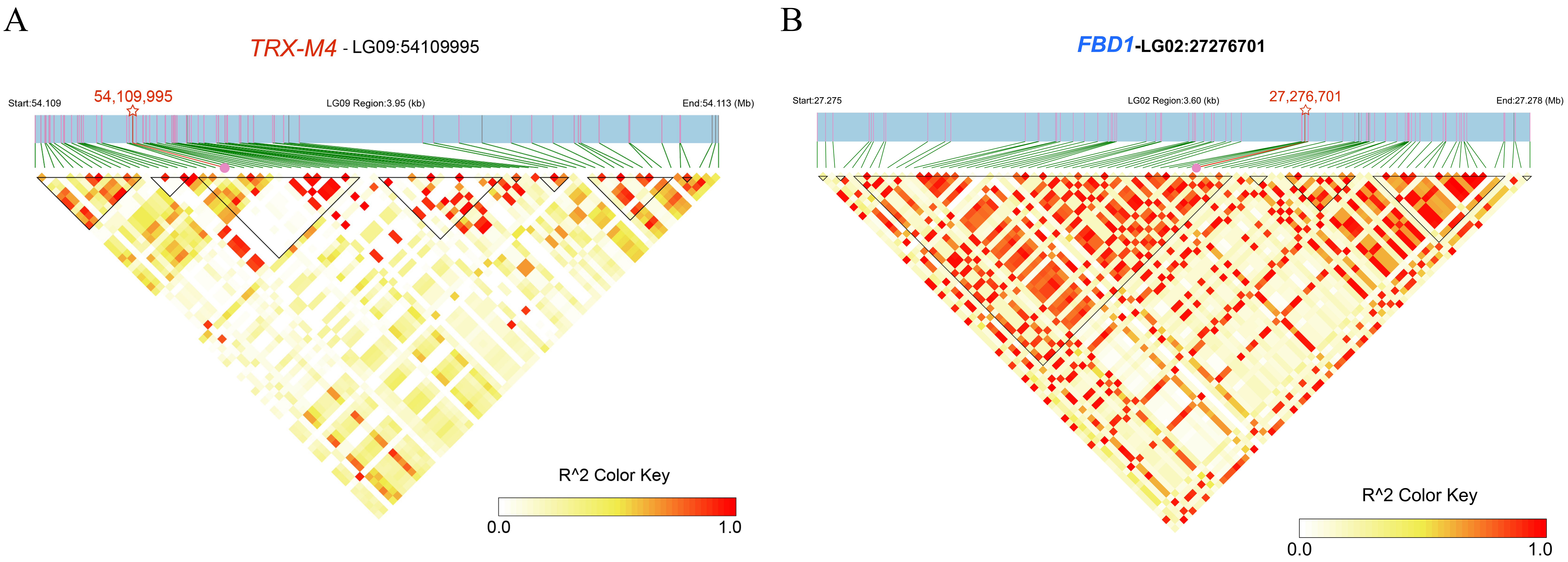

Fig. S11. LD heatmaps for genomic regions flanking the variants associated with candidate genes *TRX-M4* (A) and *FBD1* (B). The position of the adaptive variant is indicated by a five-pointed star in each panel.
