# Supplementary material for: Population and landscape genomics provide insights into the adaptive genetic variation and future climate-induced vulnerability of the endangered tree species Phoebe bournei
Source: Hortic Res. 2026 Apr 24;13(9):uhag165. doi: 10.1093/hr/uhag165 (PMC13532409; doi:10.1093/hr/uhag165)
Supplement: Supplementary file 1 [file web_material_uhag165.zip › Fig. S12.pdf]

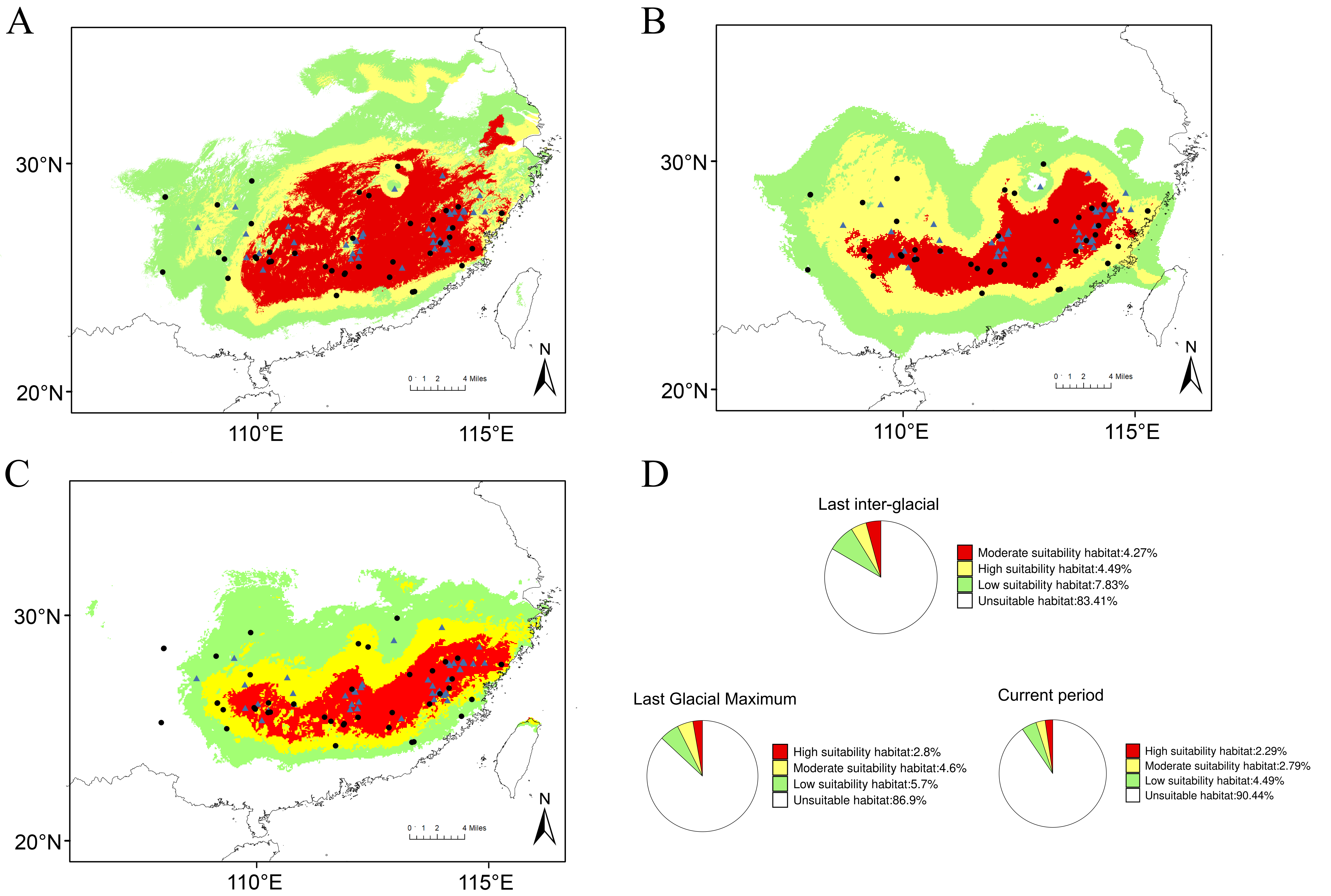

Fig. S12. Predicted natural distribution of *P. bournei* across different historical periods. (A–C) Predicted natural distribution maps of *P. bournei* during the Last Interglacial (A), Last Glacial Maximum (B), and current period (1970–2000) (C). Different colors represent habitat suitability levels: unsuitable habitat (white), low suitability habitat (green), medium suitability habitat (yellow), and high suitability habitat (red). Circles and triangles indicate unsequenced and re-sequenced populations, respectively. Blue lines represent the locations of major rivers. (D) Pie charts showing the proportions of habitat suitability classes across the three time periods.
