# Supplementary material for: Population and landscape genomics provide insights into the adaptive genetic variation and future climate-induced vulnerability of the endangered tree species Phoebe bournei
Source: Hortic Res. 2026 Apr 24;13(9):uhag165. doi: 10.1093/hr/uhag165 (PMC13532409; doi:10.1093/hr/uhag165)
Supplement: Supplementary file 1 [file web_material_uhag165.zip › Fig. S13.pdf]

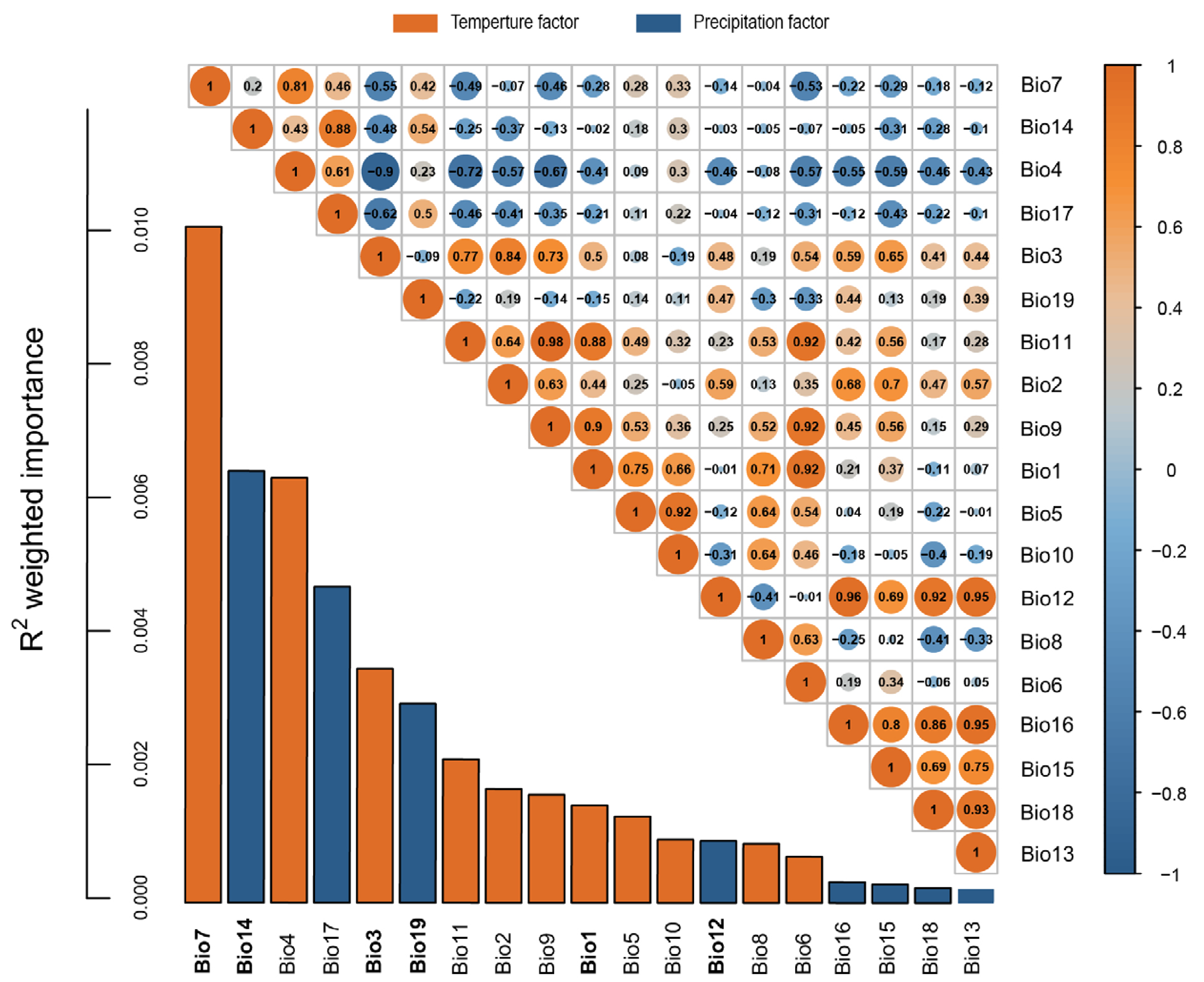

Fig. S13. Correlation and importance analysis of 19 bioclimatic variables. The upper-right triangular matrix shows pairwise Spearman correlation coefficients, with circle size and a color gradient from orange to blue representing the strength and sign of each correlation; the lower-left triangle displays the corresponding numeric values. The bar chart on the left depicts the weighted R^2 importance scores from RDA, with temperature-related variables in orange and precipitation-related variables in blue.
