# Supplementary material for: Population and landscape genomics provide insights into the adaptive genetic variation and future climate-induced vulnerability of the endangered tree species Phoebe bournei
Source: Hortic Res. 2026 Apr 24;13(9):uhag165. doi: 10.1093/hr/uhag165 (PMC13532409; doi:10.1093/hr/uhag165)
Supplement: Supplementary file 1 [file web_material_uhag165.zip › Fig. S14.pdf]

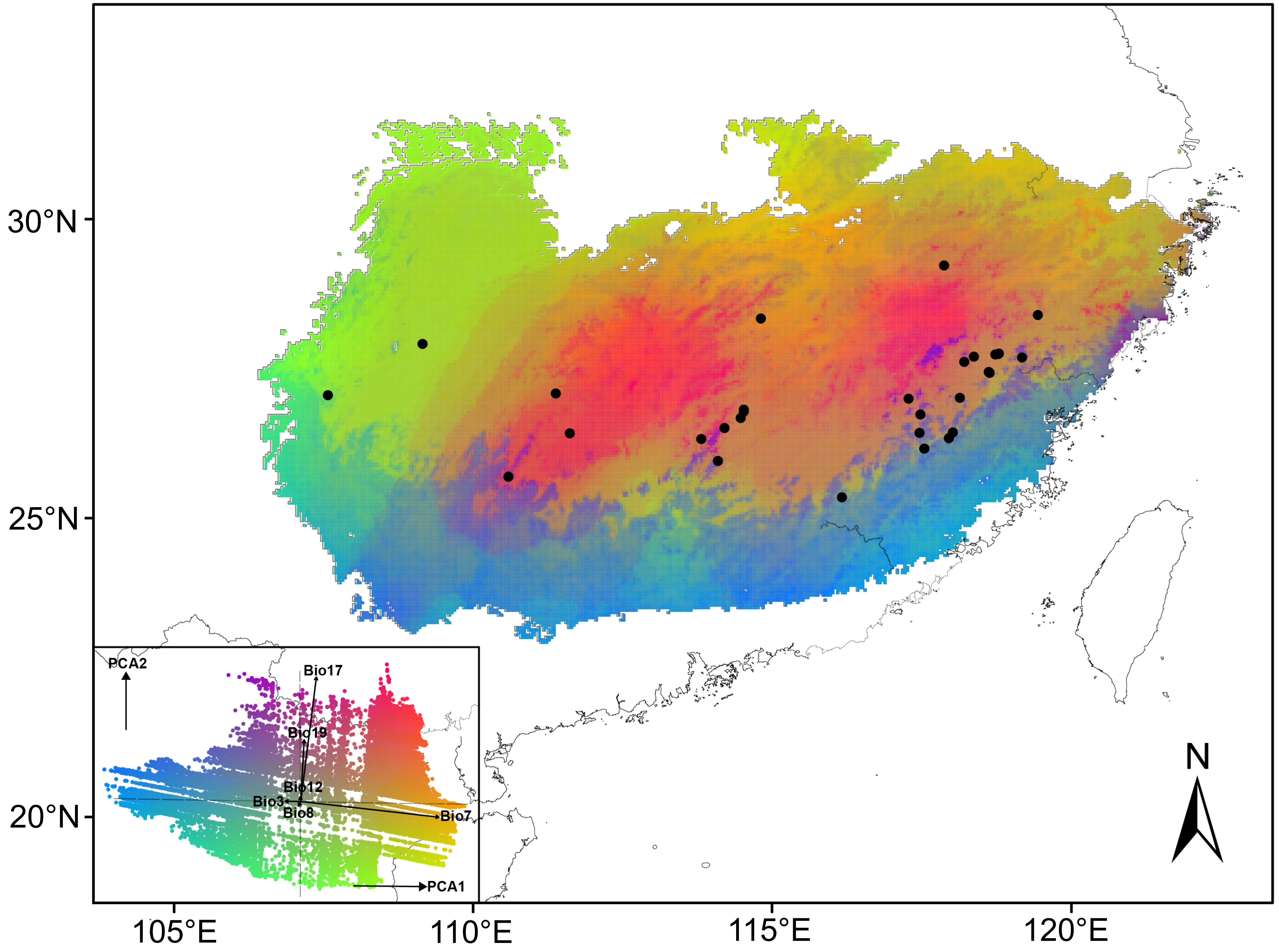

Fig. S14. Geographic projection of PCA results across the natural range of *P. bournei*. The main map employs a color gradient of combined PC1 and PC2 scores to depict regional variation in climatic factor profiles, with black dots marking sampling sites. The inset PCA biplot displays the first two principal components, and arrows indicate the direction and magnitude of loadings for six key environmental variables, illustrating their contributions to sample differentiation.
