# Supplementary material for: Population and landscape genomics provide insights into the adaptive genetic variation and future climate-induced vulnerability of the endangered tree species Phoebe bournei
Source: Hortic Res. 2026 Apr 24;13(9):uhag165. doi: 10.1093/hr/uhag165 (PMC13532409; doi:10.1093/hr/uhag165)
Supplement: Supplementary file 1 [file web_material_uhag165.zip › Fig. S15.pdf]

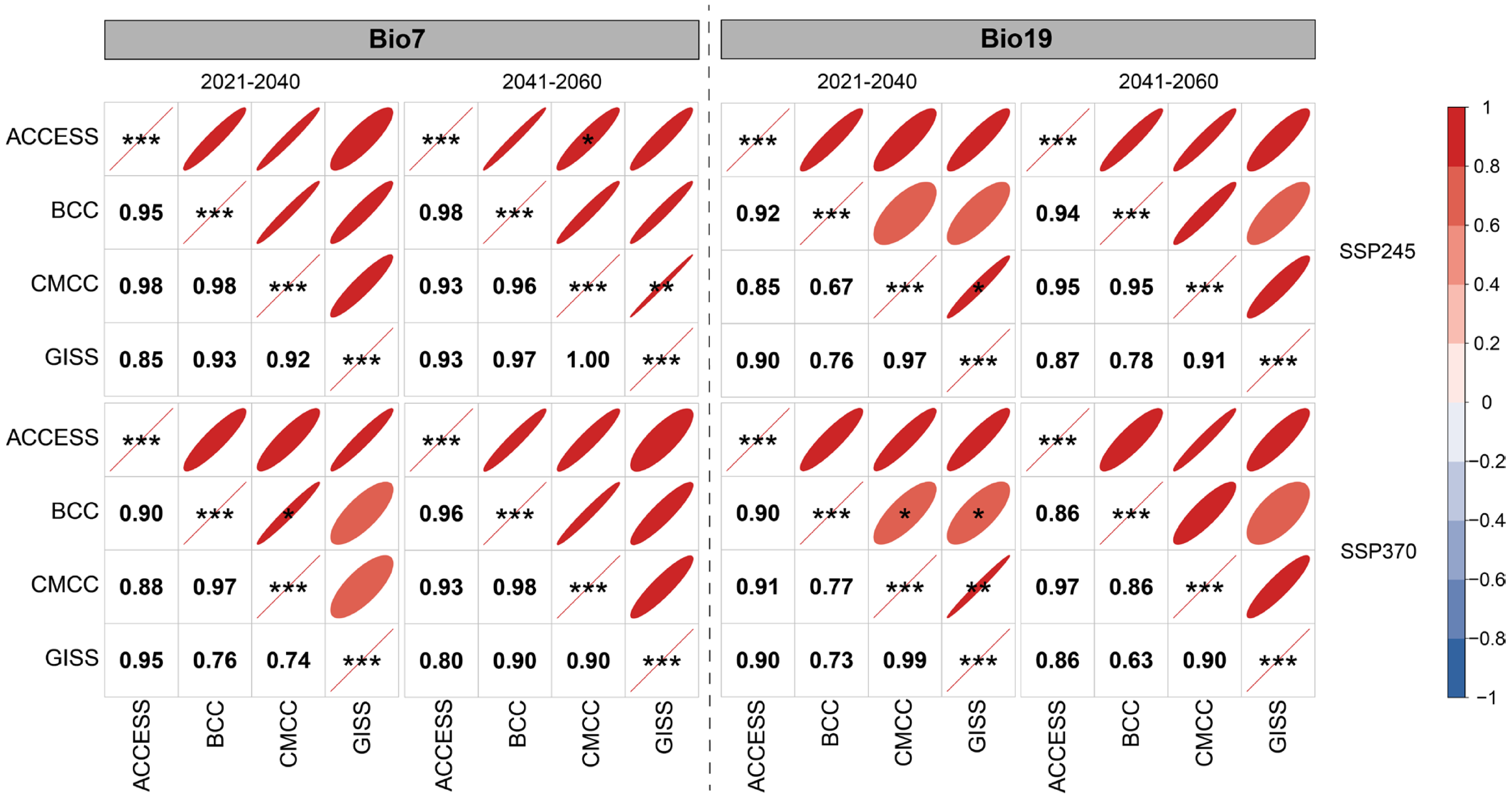

Fig. S15. Correlation matrix of RONA values estimated under four future climate models. Two-tailed Spearman correlation tests were used to assess significance; significance levels are denoted by *P < 0.05, **P < 0.01, and ***P < 0.001. Abbreviations: BCC = BCC-CSM2-MR, ACCESS = ACCESS-CM2, GISS = GISS-E2-1-G, CMCC = CMCC-ESM2.
