# Supplementary material for: Population and landscape genomics provide insights into the adaptive genetic variation and future climate-induced vulnerability of the endangered tree species Phoebe bournei
Source: Hortic Res. 2026 Apr 24;13(9):uhag165. doi: 10.1093/hr/uhag165 (PMC13532409; doi:10.1093/hr/uhag165)
Supplement: Supplementary file 1 [file web_material_uhag165.zip › Fig. S16.pdf]

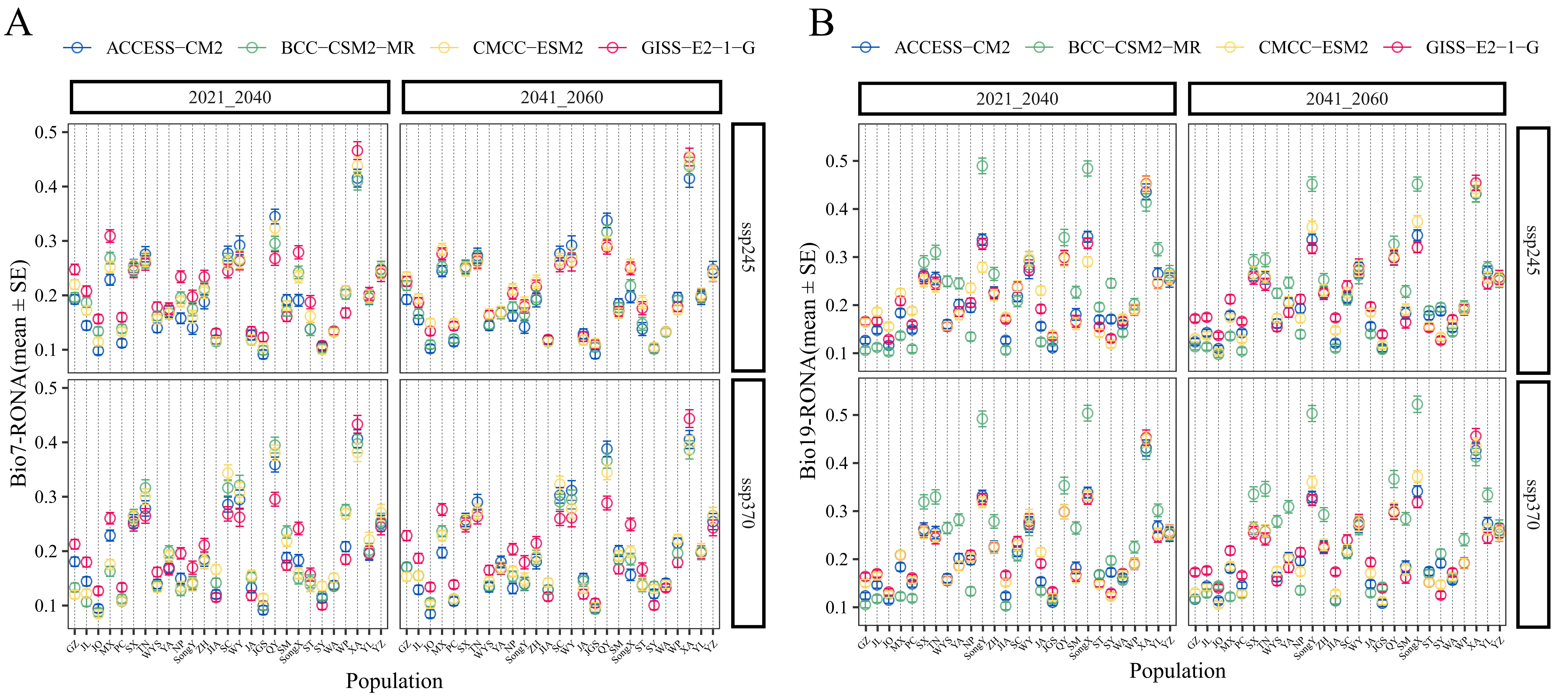

Fig. S16. Estimates of the RONA for (A) Bio7 and (B) Bio19 across 27 populations, calculated under four future climate models (color-coded) for the periods 2021–2040 and 2041–2060 under SSP245 and SSP370 scenarios. Error bars denote the standard error of the mean RONA for each model.
