# Supplementary material for: Population and landscape genomics provide insights into the adaptive genetic variation and future climate-induced vulnerability of the endangered tree species Phoebe bournei
Source: Hortic Res. 2026 Apr 24;13(9):uhag165. doi: 10.1093/hr/uhag165 (PMC13532409; doi:10.1093/hr/uhag165)
Supplement: Supplementary file 1 [file web_material_uhag165.zip › Fig. S17.pdf]

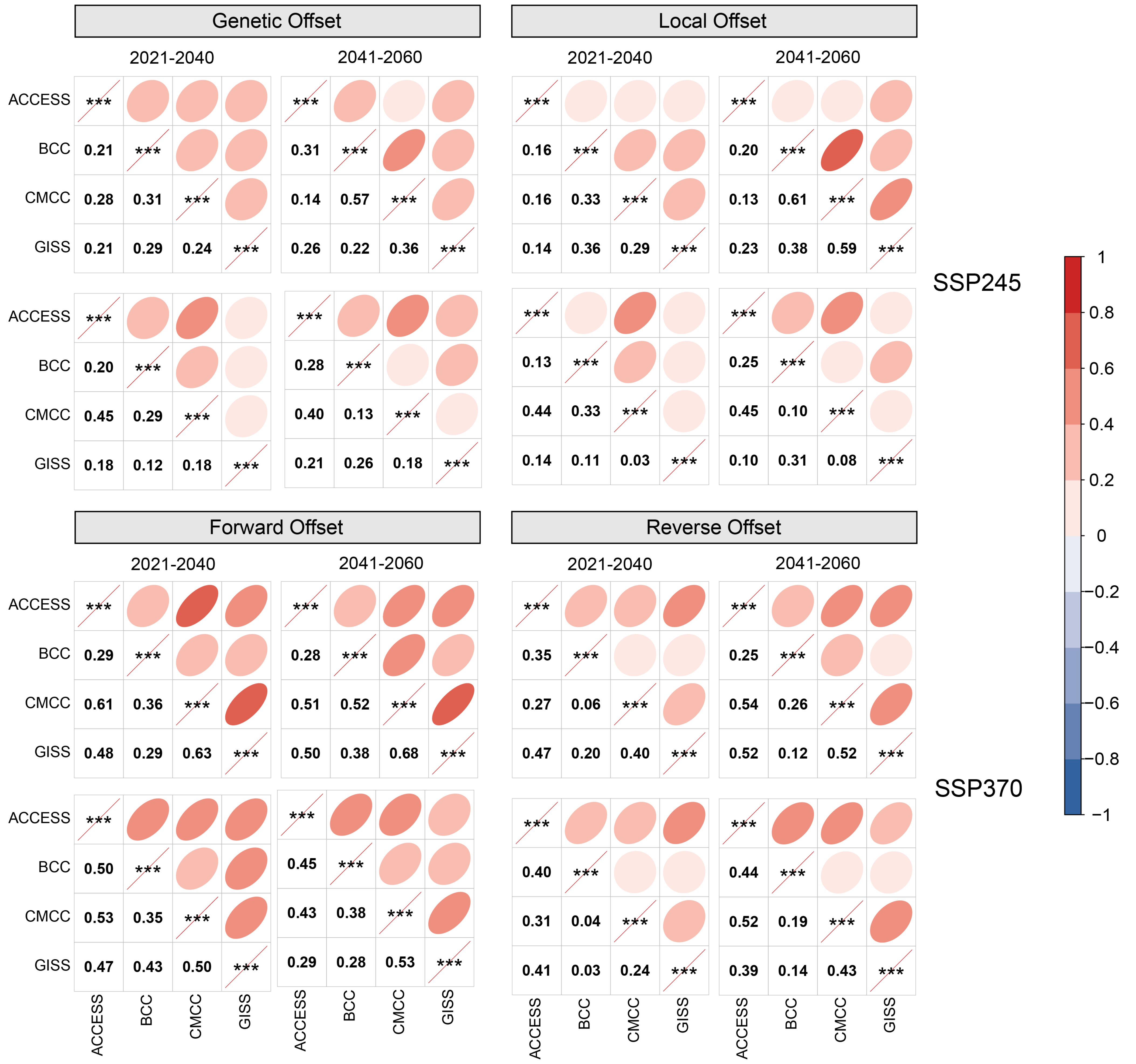

Fig. S17. Pairwise correlation matrix of GF estimates under four future climate models. Correlations are shown for the conventional Gradient Forest genetic offset and migration-adjusted local, forward, and reverse offsets across four models (BCC-CSM2-MR, ACCESS-CM2, GISS-E2-1-G, CMCC-ESM2) under two scenarios (SSP245 and SSP370) for the periods 2021–2040 and 2041–2160. Two-tailed Spearman rank correlations were used to assess significance, with *P < 0.05, **P < 0.01, and ***P < 0.001. Model abbreviations: BCC = BCC-CSM2-MR; ACCESS = ACCESS-CM2; GISS = GISS-E2-1-G; CMCC = CMCC-ESM2.
