# Supplementary material for: Population and landscape genomics provide insights into the adaptive genetic variation and future climate-induced vulnerability of the endangered tree species Phoebe bournei
Source: Hortic Res. 2026 Apr 24;13(9):uhag165. doi: 10.1093/hr/uhag165 (PMC13532409; doi:10.1093/hr/uhag165)
Supplement: Supplementary file 1 [file web_material_uhag165.zip › Fig. S3.pdf]

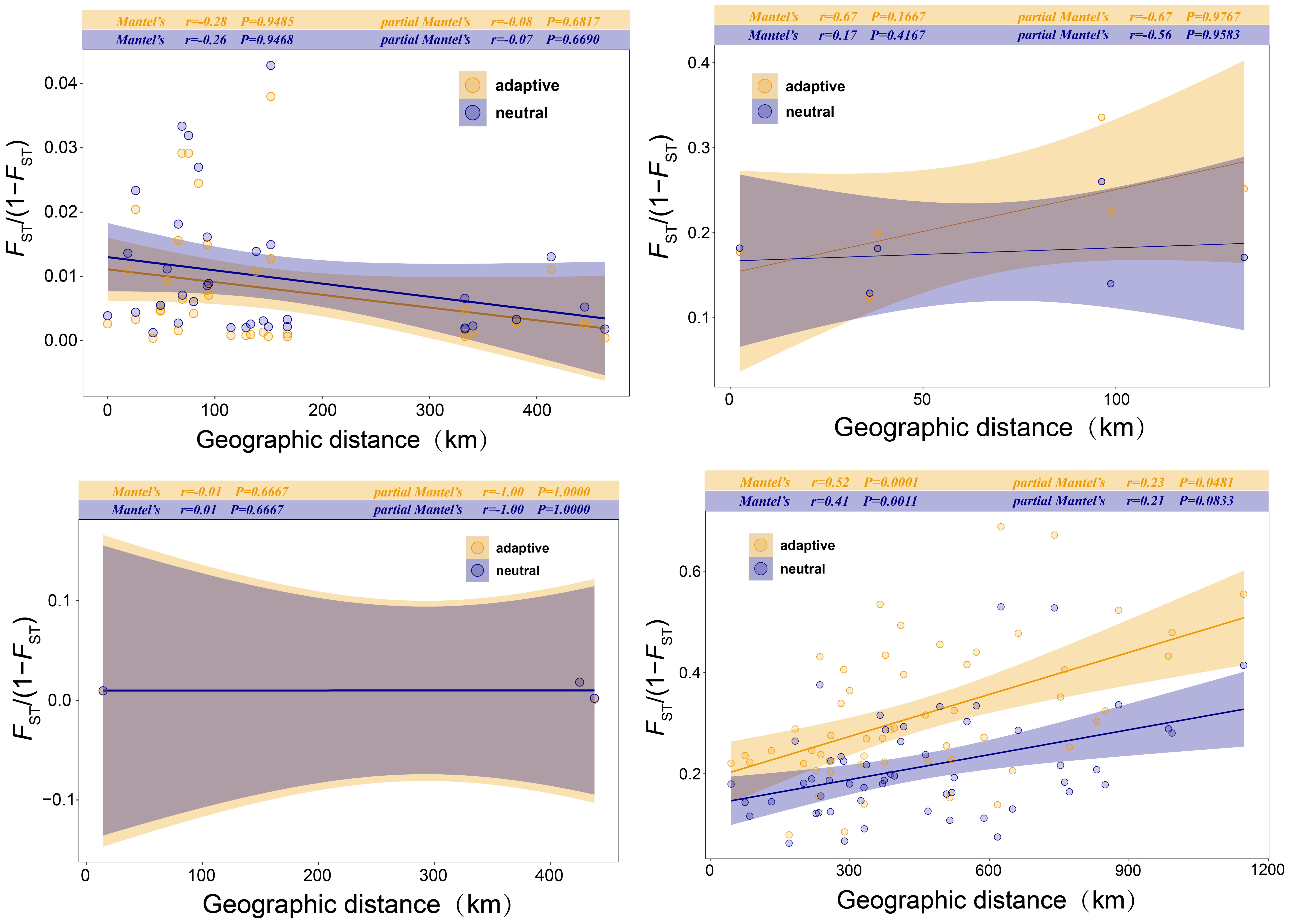


**Fig.S3.** IBD models for the four ancestral clusters. Panels (A)-(D) show IBD plots for Groups 1-4, respectively. Analyses were conducted using neutral loci (blue circles and points) and adaptive loci (yellow circles and points). Mantel tests were employed to assess the correlation between geographic distance and genetic variation, while partial Mantel tests controlled for environmental distance. Shaded areas around the regression lines indicate 95% confidence intervals.
