# Supplementary material for: Population and landscape genomics provide insights into the adaptive genetic variation and future climate-induced vulnerability of the endangered tree species Phoebe bournei
Source: Hortic Res. 2026 Apr 24;13(9):uhag165. doi: 10.1093/hr/uhag165 (PMC13532409; doi:10.1093/hr/uhag165)
Supplement: Supplementary file 1 [file web_material_uhag165.zip › Fig. S4.pdf]

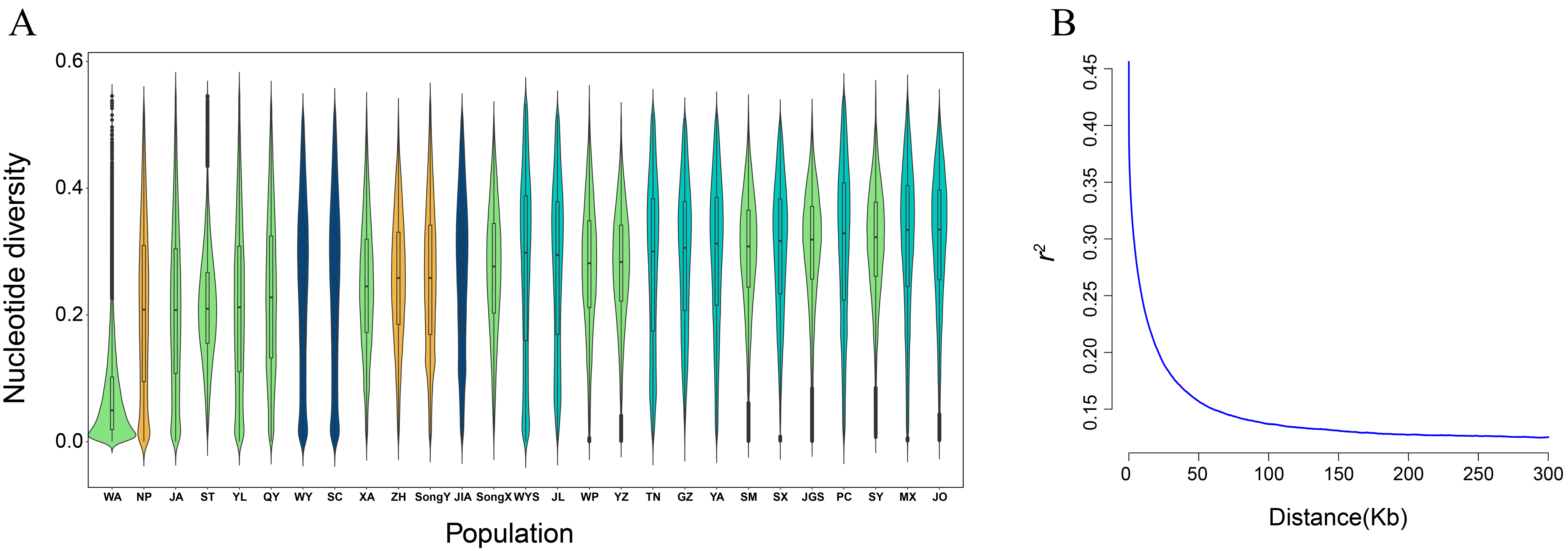

Fig. S4. (A) The distribution of nucleotide diversity across the 27 *P. bournei* populations. The populations are listed from low diversity to high, according to π medians. The box plots show the mean values and the 25th-75th percentiles (box limits) for each population.(B) The LD decay curves of the entire population, estimated using PopLDdecay.
