# Supplementary material for: Population and landscape genomics provide insights into the adaptive genetic variation and future climate-induced vulnerability of the endangered tree species Phoebe bournei
Source: Hortic Res. 2026 Apr 24;13(9):uhag165. doi: 10.1093/hr/uhag165 (PMC13532409; doi:10.1093/hr/uhag165)
Supplement: Supplementary file 1 [file web_material_uhag165.zip › Fig. S6.pdf]

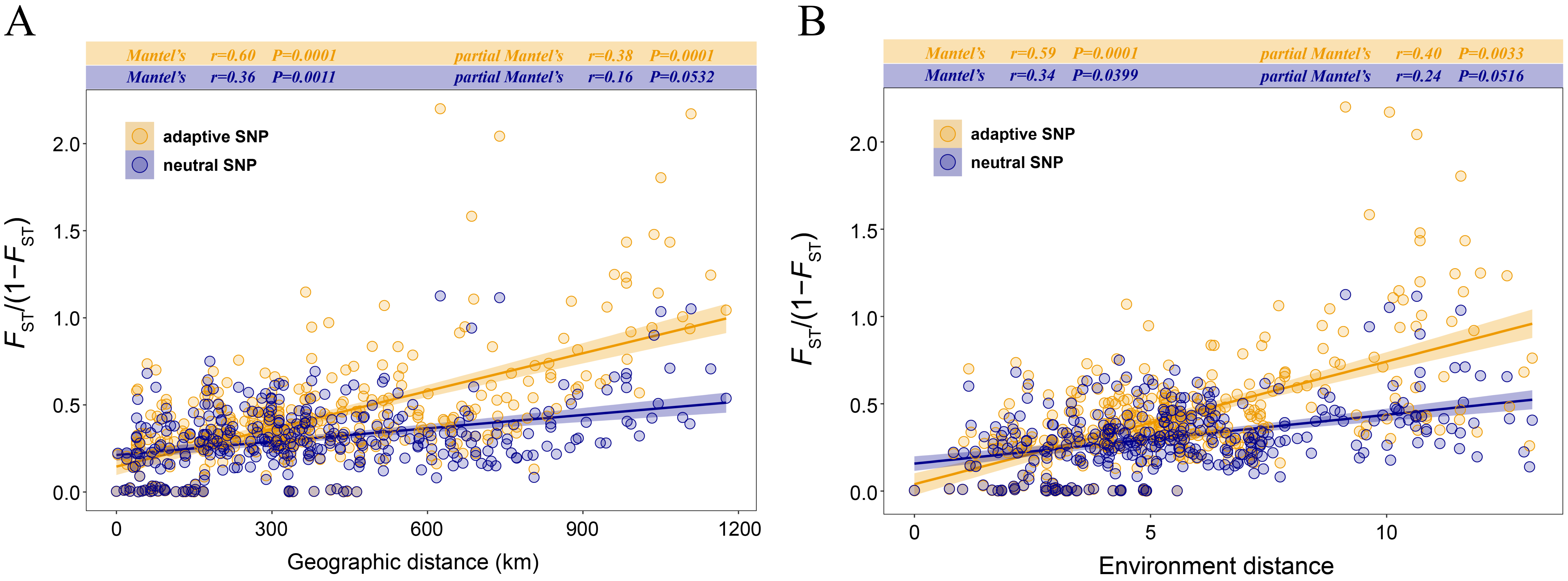

Fig. S6. IBD and IBE analyses. (A) Relationship between geographic distance and genetic distance (F_{ST} /(1–F_{ST})). (B) Relationship between environmental distance and genetic distance. Orange open circles denote adaptive loci; blue filled circles denote neutral loci. Solid lines and shaded bands represent the linear regression fits and their 95% confidence intervals, respectively. Mantel and partial Mantel test correlation coefficients (*r*) and P-values are shown in orange and blue at the top of each panel.
