# Supplementary material for: Population and landscape genomics provide insights into the adaptive genetic variation and future climate-induced vulnerability of the endangered tree species Phoebe bournei
Source: Hortic Res. 2026 Apr 24;13(9):uhag165. doi: 10.1093/hr/uhag165 (PMC13532409; doi:10.1093/hr/uhag165)
Supplement: Supplementary file 1 [file web_material_uhag165.zip › Fig. S8.pdf]

A

Adaptive variants					
Partial RDA model	Inertia	R^2(adj)	*p*(>F)	PEV	PTV
Full model:*F~clim.* + *geog.*+ *srtuct.*	115306.00	0.7288	0.001	1.00	0.75
Full model:*F~clim.*\ (*geog.*+ *srtuct.*)	24586.23	0.1487	0.001	0.21	0.16
Full model:*F~geog.*\ (*clim.* + *srtuct.*)	2017.13	0.0125	0.001	0.02	0.01
Full model:*F~struct.*\ (*clim.* + *geog*)	25442.92	0.1707	0.001	0.22	0.17
confounded climate/geography/structure	63259.69			0.55	0.41
Total unexplained	38624.03				0.25
Total inertia	153930.00				1.00

B

Neutral variants					
Partial RDA model	Inertia	R^2(adj)	*p*(>F)	PEV	PTV
Full model:*F~clim.* + *geog.*+ *srtuct.*	711756.73	0.6887	0.001	1.00	0.71
Full model:*F~clim.*\ (*geog.*+ *srtuct.*)	120109.08	0.1061	0.001	0.17	0.12
Full model:*F~geog.*\ (*clim.*+ *srtuct.*)	12414.15	0.0114	0.001	0.02	0.01
Full model:*F~struct.*\ (*clim.* + *geog*)	206993.91	0.2143	0.001	0.29	0.21
confounded climate/geography/structure	372239.58			0.52	0.37
Total unexplained	287889.26				0.29
Total inertia	999646.00				1.00

Fig. S8. A comparison of the contributions of climate, geography, and population structure to gene tic variation at adaptive (A) and neutral loci (B) based on partial RDA. The table presents total iner tia, adjusted coefficient of determination (R^2), significance (*p*(>F)), percentage of explained varian ce (PEV), and proportion of total variance (PTV) for each model.
