# Supplementary material for: Population and landscape genomics provide insights into the adaptive genetic variation and future climate-induced vulnerability of the endangered tree species Phoebe bournei
Source: Hortic Res. 2026 Apr 24;13(9):uhag165. doi: 10.1093/hr/uhag165 (PMC13532409; doi:10.1093/hr/uhag165)
Supplement: Supplementary file 1 [file web_material_uhag165.zip › Fig. S9.pdf]

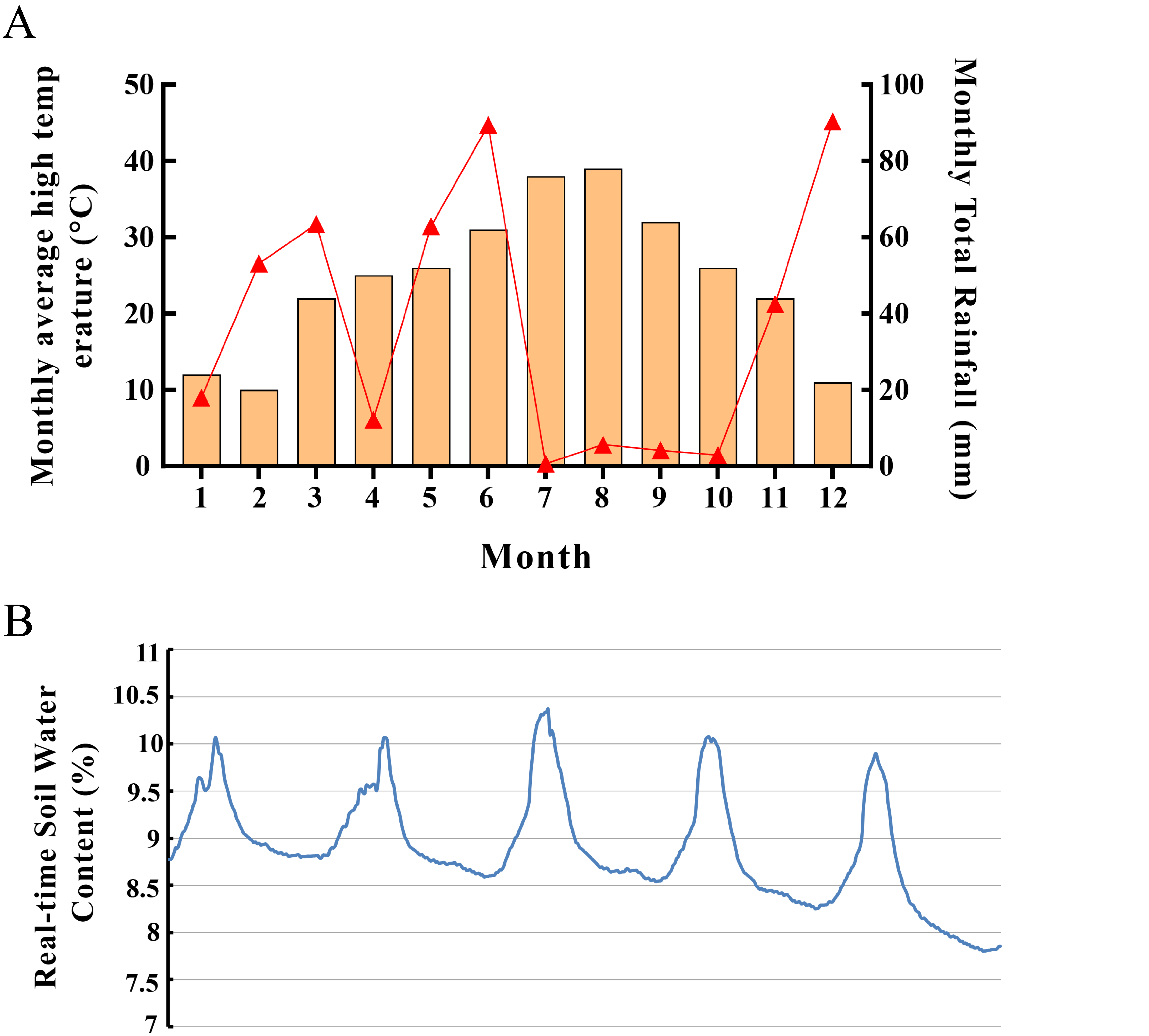

Fig. S9. Meteorological (A) and soil moisture content (B) records in the *P. bournei* germplasm bank. (A) Monthly average maximum temperature (bars) and total monthly precipitation (line) in Lishui City throughout 2022. (B) Daily variations in soil moisture content within the *P. bournei* germplasm bank from September 26 to October 1.
